# Assessing the efficacy of a low-cost air pollution monitoring device for environmental and occupational exposure assessments

**DOI:** 10.1007/s10661-025-14870-1

**Published:** 2025-12-11

**Authors:** Samuel Stowe, Riyanshi Bohra, M. J. Ruzmyn Vilcassim

**Affiliations:** 1 Department of Environmental Health Sciences, University of Alabama at Birmingham School of Public Health, 1665 University Boulevard, RPHB 530, Birmingham, AL 35233 USA; 2 The University of Arizona Zuckerman College of Public Health, Tucson, AZ USA

**Keywords:** Sensors, Particulate matter, Exposure assessment, Occupational health

## Abstract

Low-cost particulate matter (PM) sensors are increasingly used for personal and environmental air quality monitoring due to their affordability and accessibility. Recent advancements make these sensors suitable for occupational settings, but their accuracy in such settings remains uncertain. This study calibrated the AirBeam 2 and AirBeam 3 against the Thermo Scientific Personal DataRAM PDR-1500 to assess their efficacy at measuring high PM concentrations, such as those in occupational exposure settings, using engine exhaust and biomass smoke as PM sources. Laboratory calibrations were conducted using a sealed chamber. Linear and polynomial regressions assessed agreement with the PDR-1500, while breakpoint analyses identified thresholds where sensor performance shifted. Field calibrations using the AirBeam 2s evaluated real-world performance and user preferences. The AirBeam 2 exhibited a novel issue where PM₁ readings exceeded PM₂.₅ at concentrations > 50 µg/m^3^, which was corrected through reprogramming. Polynomial models outperformed linear ones for both devices and the AirBeam 3 performed better with engine exhaust than biomass smoke (linear calibration coefficients 0.192 vs 0.102, respectively), while the AirBeam 2 performed better with biomass smoke than engine exhaust (coefficients 0.323 vs 0.274, respectively). Breakpoints suggested the AirBeam 2s may be better for high concentrations, while the AirBeam 3s were more sensitive at lower concentrations. In the field, the AirBeam 2s recorded lower mean PM concentrations than the PDR-1500 and were more influenced by environmental conditions, yet participants (*n* = 9) who were recruited to perform field calibrations with both devices preferred the AirBeam. While sensor performance can vary by PM source, concentration, and environmental factors, these findings suggest AirBeams can be a useful option for preliminary occupational exposure assessments after careful calibration and validation prior to use.

**Supplementary Information:**

The online version contains supplementary material available at 10.1007/s10661-025-14870-1.

## Introduction

According to a joint report by the Health Effects Institute (HEI) and the Institute for Health Metrics and Evaluations (IHME), ambient and indoor PM_2.5_ (particulate matter less than 2.5 µm in diameter) pollution contributed to 7.8 million deaths globally in 2021, with ambient PM_2.5_ pollution alone contributing to 4.7 million of these deaths (Health Effects Institute, 2024). Exposure to particulate matter (PM) air pollution is associated with a variety of adverse health effects including cardiovascular disease, respiratory diseases and symptoms, asthma, chronic bronchitis, cancer, diabetes, exacerbation of other health conditions, and increased mortality (US Environmental Protection Agency, 2024a; Kim et al., 2015; Yang et al., 2024). Sources of PM pollution include stationary and mobile sources such as construction sites, fires, smokestacks, traffic, and photochemical reactions in the atmosphere (US Environmental Protection Agency, 2024b). In the USA, ambient PM concentrations have traditionally been monitored by the Environmental Protection Agency (EPA) using advanced and centrally located stationary PM monitoring instruments (Koehler & Peters, 2015). However, these stationary monitors cannot account for personal level spatiotemporal variations in PM concentrations, and instead, personal monitoring of PM is regarded as the best representative measurement of an individual’s exposure (Dai et al., 2020; Gorai et al., 2018; Koehler & Peters, 2015; Larkin & Hystad, 2017; Nerriere et al., 2005; Vilcassim & Thurston, 2023; Wang et al., 2015a, b). Until around the 2010s, the personal monitors available on the market were advanced, relatively expensive in the range of $5,000 to $10,000, and required training to operate, with examples including the Thermo Scientific Personal Data RAM PDR-1500 (Waltham, Massachusetts) and the TSI DustTrak II Aerosol Monitor (Shoreview, Minnesota). However, due to a combination of increased public interest on air pollution and advancements in affordable technology, companies have begun producing low-cost personal PM monitoring sensors and devices which are inexpensive, lightweight, and easy to use (Koehler & Peters, 2015; Kumar et al., 2015; Snyder et al., 2013), causing them to now be widely used by the public, non-profit organizations, interest groups, and even some researchers who have used them to monitor personal exposures and environmental PM concentrations (Anastasiou et al., 2022; Lim et al., 2019; Morawska et al., 2018).

Developing rapidly, these low-cost PM sensors may have the potential to substitute the more advanced and industry standard personal PM monitoring devices in some settings, which would make them a viable option for wider use, such as in occupational settings. First, they are significantly cheaper than the more advanced industry standard monitors, with low-cost air quality monitors costing only a few hundred dollars per unit as compared to the industry standard personal monitors which cost five to ten thousand dollars per unit, allowing for more workers to be monitored without incurring high costs. Second, they are lightweight and smaller in size than the industry standard personal monitors, making them less cumbersome to use during a personal monitoring session and less likely to impact workers’ behavior. Third, they are easy to use and require little training to begin taking measurements. Lastly, many low-cost air quality monitors stream their temperature, humidity, PM concentration (broken down further by size fraction into PM_1_, PM_2.5_, and PM_10_), and location data via wireless connections to phones and/or servers, making it easy to retrieve the data and share it to crowdsourcing platforms.

People working in occupational settings are often exposed to high and potentially hazardous concentrations of PM, making it important to monitor their exposures to protect their health (Fang et al., 2010; Garcia et al., 2013; Sousan et al., 2016). Currently, the standard for personal monitoring of PM in occupational settings is to use either gravimetric methods or advanced nephelometric devices, such as the Thermo Scientific Personal DataRAM PDR-1500 and the TSI DustTrak II Aerosol Monitor, which have been proven to be accurate at measuring PM concentrations in real time (Cambra-López et al., 2015; Halterman et al., 2017; Kim et al., 2004; Marto et al., 2021). However, the high cost per unit (typically in the range of $5,000–$10,000) of these devices limits the number of units that can be used to measure workers’ exposures (Sousan et al., 2016).

Low-cost PM sensors measure PM concentrations using light scattering techniques, similar to the ones used in the previously mentioned advanced nephelometric devices, but do so at a fraction of the cost, making them a potential option for scaling up occupational exposure assessments (Liu et al., 2017; Sousan et al., 2016). Additionally, their ability to stream their data, including location information, can be combined with worker exposure data and activity diaries for more temporally and spatially accurate exposure assessments. Prior studies comparing these low-cost PM sensors to the more advanced personal monitors in laboratory settings have shown that these devices are capable of measuring PM concentrations with high correlation to the more advanced personal monitors, within a certain range (Karagulian et al., 2019; Kaur & Kelly, 2023a, 2023b; Kelly et al., 2017; Kim et al., 2019; Levy Zamora et al., 2019; Lim et al., 2019; Liu et al., 2017). In addition, several recent studies, including some by the EPA, have shown that these low-cost monitors can be reliably used for monitoring ambient PM concentrations in the field (Bulot et al., 2019; Jiao et al., 2016; Kelly et al., 2017; Levy Zamora et al., 2019; Lim et al., 2019; Williams et al., 2014; Zheng et al., 2018). However, it is worth noting that the range of PM concentrations in these studies was usually below 100 µg/m^3^. Though, one recent study that evaluated the performance of several low-cost sensors during high-concentration dust events in Salt Lake Valley demonstrated that these sensors were able to capture temporal patterns and performed relatively well when compared against reference monitors (K. Kaur & K. E. Kelly, 2023). Furthermore, several recent studies have also begun to test the performance of low-cost sensors in high-concentration occupational environments such as mines. These studies have shown that, with proper calibration, sensors can provide reasonable agreement with reference instruments for coal dust and other particulate sources, suggesting their feasibility for occupational exposure monitoring (Amoah et al., 2023; Penchala et al., 2025; Zaid et al., 2024).

However, prior studies have demonstrated limitations to these low-cost PM sensors such as plateauing, reliability issues, and inter-instrument variability (Feinberg et al., 2018; Kelly et al., 2017; Vercellino et al., 2018). Compared to the more expensive industry standard monitors, the sensors in low-cost PM sensors tend to be less technologically advanced and therefore struggle to match the accuracy and precision of their industry standard counterparts. Moreover, concerns have been raised regarding how the chemical composition of PM might impact the light scattering capabilities of these sensors and consequently affect their ability to precisely measure PM concentrations. Therefore, these devices must first be validated before they can be used in occupational exposure assessments, where PM concentrations are typically significantly higher than environmental levels. Fortunately, prior studies have shown that calibration factors that improve the accuracy of the data they collect can be developed by lab testing and calibrating these devices prior to their use (Badura et al., 2019; Lee et al., 2020; Y. Wang et al., 2015a, 2015b; Zusman et al., 2020). Furthermore, while other studies have investigated whether low-cost PM sensors can be used in occupational settings, research remains limited and therefore further investigation is necessary as these sensors have the potential to democratize air pollution exposure assessments by making the necessary equipment available to monitor more workers at a lower cost.

Given the above, the objectives of this study were to investigate how low-cost sensors behave when exposed to a wide range of PM concentrations, whether the PM’s chemical composition affects these devices’ ability to accurately measure PM concentrations, and if a selected brand of low-cost PM monitoring device—the AirBeam (Brooklyn, New York)—can be reliably used to measure PM concentrations typically seen in occupational settings. We specifically examined particles from biomass and gasoline combustion, as they are common sources of PM_2.5_.

## Methods

A series of lab calibrations and field calibrations were completed on two “generations” of AirBeams (a low-cost sensor device), the AirBeam 2 and the AirBeam 3. Both generations of AirBeams were calibrated against a more advanced and widely used nephelometric PM monitoring device, the Thermo Scientific Personal DataRAM PDR 1500, outfitted with a PM_2.5_ cut-point physical cyclone operated at 1.52 L/min. The factory calibrated PDR 1500 was zeroed using particle-free air before each calibration run. Both the AirBeam 2 and 3 use a Plantower PMS 7003 laser light scattering particle sensor which uses an internal algorithm to differentiate between particle sizes. Data from the PDR 1500 was downloaded directly via USB to a computer, and data from the AirBeams were streamed to a cellphone where they were then downloaded to a computer. Linear and polynomial regressions were generated for each AirBeam calibration for both the lab and field calibrations (described below), from which the correlation coefficients, *R*^2^ values, and calibration curves were derived.

The lab calibrations and field calibrations of the AirBeam 2 s were performed separately from the AirBeam 3 in the first phase of this study, as the AirBeam 3 had not yet been released at the initiation of this study. Once the AirBeam 3 was released, a second phase of the study was included to lab calibrate the AirBeam 3s and compare them to the AirBeam 2 devices. For the field calibrations, participants working in a variety of occupational environments were recruited to perform field calibrations of the AirBeam 2 vs the PDR 1500 while they performed their normal work, after which they were provided with a questionnaire on device preference.

### Lab calibration of the AirBeam devices

In the first phase, the AirBeam 2 devices (*n* = 10) were calibrated by placing 2 to 6 AirBeams into a sealed plastic chamber, which kept temperature and humidity consistent, with one PDR 1500 unit. The monitors were then exposed to the exhaust of a gasoline leaf blower engine. The AirBeams and the PDR 1500 were run for approximately 60–90 min, where they simultaneously recorded 1-min average PM concentrations in the chamber. The devices were then left in the chamber to take measurements until the PDR 1500 measured PM concentrations at or near 0 µg/m^3^, which typically occurred after around 1 to 1.5 h.

Next, in the second phase, the AirBeam 3 units (*n* = 3) were calibrated by placing three AirBeam 3s and three to five AirBeam 2s into a sealed plastic chamber with two PDR 1500s. The AirBeams and the PDR 1500s were then run simultaneously to record the 1-min average PM concentrations in the chamber before being exposed to either the exhaust of a gasoline leaf blower engine or biomass smoke from burning incense. After exposure, the chamber was closed and sealed, and the devices were then left to take measurements, as described previously, until the PDR 1500s measured PM concentrations at or near 0 µg/m^3^.

After each calibration run, the data from each of the devices was downloaded onto a computer where it was then analyzed using R version 4.3.1. The data from the AirBeams were then synchronized with the data from the PDR 1500 by aligning the timestamps on the data files from each device. Once all the data from a calibration run had been synchronized, calibration curves ranging from 0 up to 1000 µg/m^3^ were generated using linear regression and polynomial regression models. The calibration coefficient and *R*^2^ value from the linear and polynomial regression models for each unit were then recorded and linked to their corresponding unit. The calibration coefficient is the coefficient of the slope in the calibration equation and shows how much the AirBeam’s PM_2.5_ concentration changes for every 1 µg/m^3^ change in the PDR 1500’s PM_2.5_ concentration. The *R*^2^ value tells you how much of the variability in the dependent variable (AirBeams) is described by the independent variable (PDR 1500). Due to concerns regarding overfitting the data, especially at higher PM concentrations where data was sparse, we avoided using higher-order polynomial models and instead chose to use quadratic models given they provided a noticeable improvement over linear models while remaining stable and interpretable. Additionally, reverse calibration coefficients, which can be applied to the AirBeam’s measurements to get a more accurate approximation of the true PM concentration when measuring at high occupational concentrations, were calculated for both generations of AirBeams when measuring both types of aerosols (engine exhaust and biomass smoke). The reverse calibration coefficients were calculated by flipping the regression models so that the AirBeam was on the *X* axis and the PDR1500 was on the *Y* axis. Then, segmented regression plots were generated for both generations of AirBeams using data from the lab calibrations of the AirBeam 3s to perform breakpoint analyses. To identify points at which sensor performance began to deviate from linearity, we used piecewise linear regression with a single breakpoint for each AirBeam device. This was implemented using the “segmented” package in R, which fits two linear segments joined at a breakpoint estimated from the data. An initial guess (10 µg/m^3^) was provided, but the final breakpoint location was optimized by the algorithm. The breakpoint analyses were performed on all AirBeam 3s and AirBeam 2s in each calibration for both the engine exhaust and biomass smoke lab calibrations to determine the concentrations above which there was a notable change in sensor behavior/performance compared to each PDR 1500. Finally, we performed tenfold cross validations comparing linear vs quadratic models for the AirBeam 2s and 3s in both the engine exhaust and biomass smoke calibrations to provide a clear recommendation about which models should be used above and below the breakpoints.

#### Reprograming the AirBeam 2’s internal algorithm

Each AirBeam 2 device’s (*n* = 10) internal algorithm/calibration equation was restored to the Plantower sensor’s default algorithm after some preliminary analyses revealed that the measured PM_1_ and PM_2.5_ distribution curves were impacted by the algorithm that was installed later by the device manufacturer (Habitat Map, Brooklyn, New York) (Supplementary Figure S1). The rule we employed to identify this measurement error was that the ratio of PM_1_ to PM_2.5_ should never be greater than 1 or, in other words, anytime that the AirBeam 2 read the PM_1_ concentration as being higher than the PM_2.5_ concentration at a given time point, we considered that measurement to be incorrect. We considered these measures to be incorrect because it is impossible for the PM_1_ concentration to be higher than the PM_2.5_ concentration because, by definition, PM_2.5_ includes PM_1_. The definition of PM_2.5_ is “fine inhalable particles, with diameters that are generally 2.5 µm and smaller” (US Environmental Protection Agency, 2024b), while the definition of PM_1_ is “particulate matter less than 1 micron in size” (IQAir, 2021). As you can see from these definitions, PM_2.5_ includes any particles 2.5 microns and smaller and therefore includes PM_1_, which is particulate matter 1 micron and smaller. This measurement error was seen in all 10 AirBeam 2s prior to being reprogrammed at PM_2.5_ concentrations of 50 µg/m^3^ and above. To reprogram the AirBeam 2s’ internal algorithms, each AirBeam 2 was connected to a computer via USB and placed into reprogramming mode, after which the internal algorithms were manually reprogrammed by deleting the “new” algorithms programmed into each sensor by the AirBeam’s manufacturer and replacing them with the default algorithms that the Plantower sensors were originally programmed with. Detailed instructions on how we reprogrammed the AirBeam 2s to use the Plantower sensor’s default internal algorithm are included in the supplemental section. These reprogrammed AirBeam 2 units were then calibrated against a PDR1500 using the same leaf blower exhaust as described above.

### Field calibration of the AirBeam 2s

For the field calibrations of the AirBeam 2s, nine participants (*n* = 9) working in specific occupational environments, including construction, landscaping, carpentry, and heavy machine operating, were recruited to conduct calibrations in occupational environments. Participants were recruited primarily from their worksites, where they were given a brief explanation of the study before the research team obtained their informed consent. University of Alabama at Birmingham (UAB) Institutional Review Board (IRB) approval was obtained prior to enrolling participants for the study (UAB IRB# 300004373). Informed consent was received from each participant prior to their participation, after which they were officially enrolled in the study. Participants received compensation after each session they completed.

The field calibrations of the AirBeam 2s were completed in two successive sessions per participant. In the first session, participants wore one AirBeam and one PDR 1500 separately for 30 min each while they did their normal work. In the second session, participants wore both devices simultaneously for 30 to 45 min while they completed their normal work. After the participants completed both sessions wearing each device separately and then together, they were given a survey to complete. The survey asked them questions about what they did for work, which air quality monitor they preferred to wear during work, which monitor they felt was more accurate, and whether they had ever worn an air quality monitor before. The purpose for completing the field calibration in two sessions was so that we could evaluate which device participants preferred to wear while working in the first session and then compare each device’s simultaneous measurements in the second session.

After each calibration was completed, the data from each device was downloaded onto a computer where it was analyzed using R version 4.4.3. Each AirBeam’s measurements were averaged into 1-min intervals, which were synched to the PDR 1500’s data by aligning the timestamps on the data files from each device. Then, calibration curves ranging from 0 up to 500 µg/m^3^ were generated for each device using linear and polynomial regression models. Then, the calibration equations and *R*^2^ values from both the linear and polynomial regression models were recorded and linked to their corresponding units. Finally, due to missing data being a consistent issue in the field calibrations of the AirBeam 2s, a sensitivity analysis where key calibration metrics were re-estimated using only field calibrations with greater than or equal to 80% data completeness was performed to determine if missing data had a significant effect on our results and therefore our conclusions.

To help minimize missing data during field measurements, users can follow the Quality Assurance/Quality Control checklist we have provided in the supplemental section. This checklist outlines essential steps to avoid data loss such as ensuring devices are fully charged, maintaining stable data connections, following recommended co-location times during calibrations, and routinely recalibrating sensors.

## Results

### Post reprogramming AirBeam 2 lab calibrations

Time series graphs (Supplementary Figure S1) generated from AirBeam 2 measurements taken after reprogramming the sensors’ algorithms back to their default equations showed that the AirBeam 2s were consistently measuring the PM_2.5_ concentrations as being higher than the PM_1_ concentrations, demonstrating that reprogramming the AirBeam 2’s internal algorithms had successfully addressed the issue seen during the first round of lab calibrations where the AirBeam 2s consistently measured PM_1_ concentrations as being higher than PM_2.5_ due to an error in their manufacturer programmed internal algorithms. However, it was also shown that after reprogramming their internal algorithms, the AirBeam 2s’ PM_2.5_ measurements consistently tracked very closely with their PM_10_ measurements (Supplementary Figure S1). Furthermore, regression models generated from the reprogrammed AirBeam 2’s PM_2.5_ measurements showed that, unlike the pre-reprogramming AirBeams, they recorded erroneous measurements at high PM concentrations (typically above 700 µg/m^3^). As a result, the reprogrammed AirBeam 2’s had a lower *R*^2^ value with the PDR 1500’s PM_2.5_ measurements than the pre-reprogramming AirBeams until these erroneous measurements were removed (Table 1 and Supplementary Table S1). All erroneous measurements were identified both visually and statistically using the interquartile range (IQR) method as measurements where an error had clearly occurred in the AirBeam 2. An example of an erroneous measurement was when AirBeam #0138 randomly measured the PM_2.5_ concentration as 4 µg/m^3^ while the PDR 1500 measured the PM_2.5_ concentration as 658 µg/m^3^, before immediately returning to normal in the next measurement where the AirBeam measured the PM_2.5_ concentration as 874 µg/m^3^ while the PDR 1500 measured the concentration as 673 µg/m^3^. It is worth noting that erroneous measurements were infrequent though with no more than 4 erroneous measurements per calibration.

**Table 1:** Calibration coefficients and *R*^2^ values for each AirBeam 2’s PM_2.5_ measurements before and after reprogramming, derived from linear and polynomial regression models

AirBeam #	Linear model				Polynomial model					
	Pre-reprogram coefficient (95% CI)	Pre-reprogram ***R***^2^	Post-reprogram coefficient (95% CI)	Post-reprogram ***R***^2^	Pre-reprogram ***X***^2^ coefficient (95% CI)	Pre-reprogram ***X*** coefficient (95% CI)	Pre-reprogram ***R***^2^	Post-reprogram ***X***^2^ coefficient (95% CI)	Post-reprogram ***X*** coefficient (95% CI)	Post-reprogram ***R***^2^
014D	0.42 (0.40–0.45)	0.98	1.14 (1.09–1.18)	0.98	−8.8e-5 (−1.8e-4 to -2.6e-6)	0.49 (0.42–0.56)	0.985	−0.0007 (−0.0008 to -0.0007)	1.61 (1.57–1.66)	0.998
0114	0.33 (0.17–0.49)	0.41	1.13 (1.05–1.21)	0.93	−0.0016 (−0.0018 to -0.0014)	1.52 (1.35–1.69)	0.946	−0.0011 (−0.0012 to -0.0010)	2.00 (1.94–2.06)	0.996
014B	0.39 (0.38–0.41)	0.97	1.45 (1.40–1.50)	0.98	−0.0002 (−0.0003 to -0.0002)	0.59 (0.57–0.61)	0.997	−0.001 (−0.001 to -0.001)	2.04 (2.01–2.07)	0.999
0138	0.40 (0.39–0.42)	0.97	1.47 (1.40–1.55)	0.96	−0.0002 (−0.0003 to -0.0002)	0.60 (0.58–0.62)	0.995	−0.0013 (−0.0014 to -0.0012)	2.23 (2.18–2.29)	0.998
030B	0.41 (0.38–0.43)	0.95	1.37 (1.29–1.45)	0.95	−0.0003 (−0.0004 to -0.0002)	0.67 (0.64–0.70)	0.995	−0.0014 (−0.0015 to -0.0013)	2.21 (2.13–2.29)	0.995
0154	0.43 (0.40–0.45)	0.97	1.36 (1.33–1.39)	0.99	−0.0003 (−0.0004 to -0.0002)	0.65 (0.63–0.67)	0.998	−0.0006 (−0.0007 to -0.0005)	1.68 (1.61–1.74)	0.997
030A	0.35 (0.33–0.37)	0.95	0.96 (0.73–0.95)	0.87	−0.0003 (−0.0004 to -0.0002)	0.59 (0.57–0.61)	0.995	−0.0012 (−0.0011 to -0.0008)	1.98 (1.64–1.95)	0.979
0161	0.36 (0.34–0.38)	0.95	1.17 (1.04–1.30)	0.9	−0.0003 (−0.0004 to -0.0002)	0.59 (0.56–0.61)	0.995	−0.0015 (−0.0014 to -0.0012)	2.23 (2.07–2.39)	0.984
032F	0.39 (0.37–0.42)	0.96	1.65 (1.52–1.78)	0.95	−0.0003 (−0.0004 to -0.0002)	0.63 (0.61–0.64)	0.998	−0.0023 (−0.0025 to -0.0021)	2.79 (2.71–2.87)	0.998
0354	0.42 (0.40–0.44)	0.94	1.25 (1.19–1.32)	0.97	−0.0004 (−0.00038 to -0.00032)	0.71 (0.69–0.74)	0.995	−0.0012 (−0.0013 to -0.0012)	2.01 (1.96–2.06)	0.998
Mean [SD]	0.39 [0.033]	0.91 [0.173]	1.3 [0.203]	0.95 [0.038]	−0.0004 [0.0004]	0.70 [0.28]	0.990 [0.015]	−0.0012 [0.0005]	2.08 [0.31]	0.994 [0.007]

After removing these erroneous measurements, linear regression models showed that there was a strong linear relationship and a high *R*^2^ value between the reprogrammed AirBeam 2s’ and PDR 1500’s measurements up to 1000 µg/m^3^. Polynomial regression models generated from these same measurements showed a higher *R*^2^ value between the reprogrammed AirBeam 2s’ and PDR 1500’s measurements, indicating that the polynomial regression models were a better fit (Table 1). Lastly, average weighted calibration coefficients were calculated for the reprogrammed AirBeam 2s’ PM_1_ and PM_2.5_ measurements using the calibration coefficients and weights listed in Supplementary Table 2. The average weighted coefficients (polynomial) for PM_1_ and PM_2.5_, respectively, were 1.37 and 2.49.

### Performance of AirBeam 3 s against the AirBeam 2 s and a PDR 1500 data RAM

**Fig. 1:**
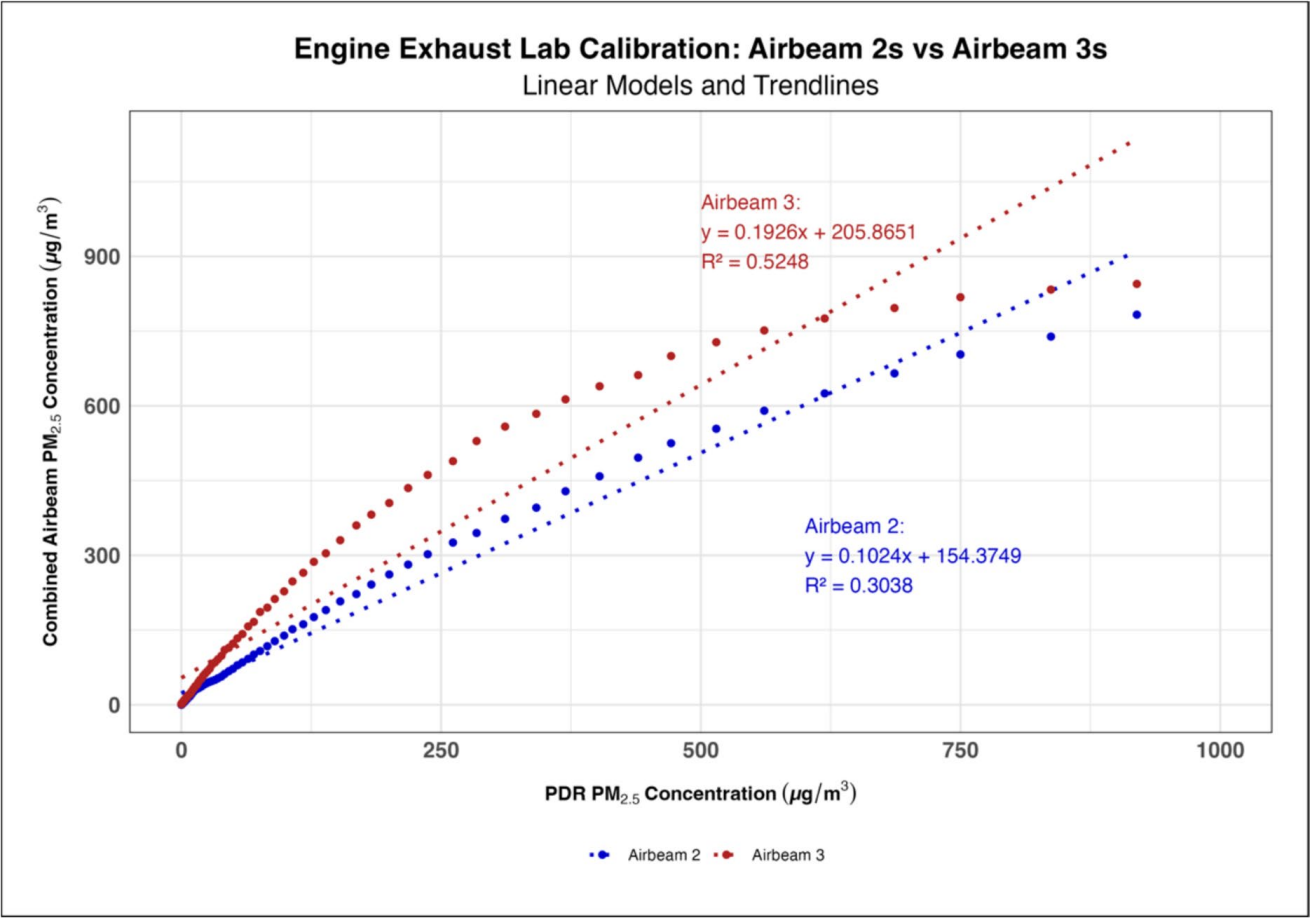
Linear regression model showing how the AirBeam 3s had a higher calibration coefficient on average compared to the AirBeam 2s when measuring engine exhaust

**Fig. 2:**
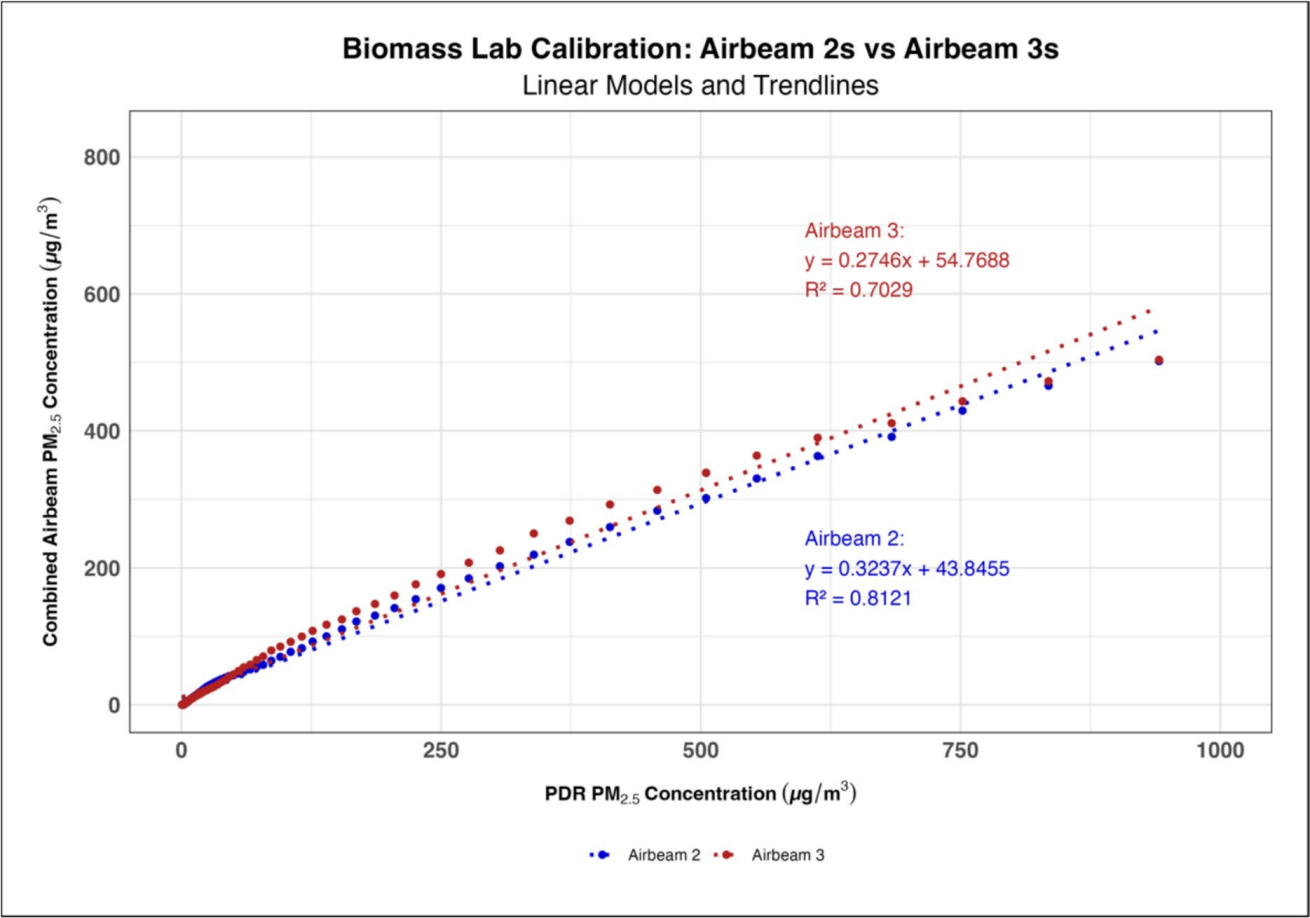
Linear regression model showing how the AirBeam 3s had slightly lower calibration coefficients and *R*^2^ values than the AirBeam 2s when measuring biomass smoke

**Fig. 3:**
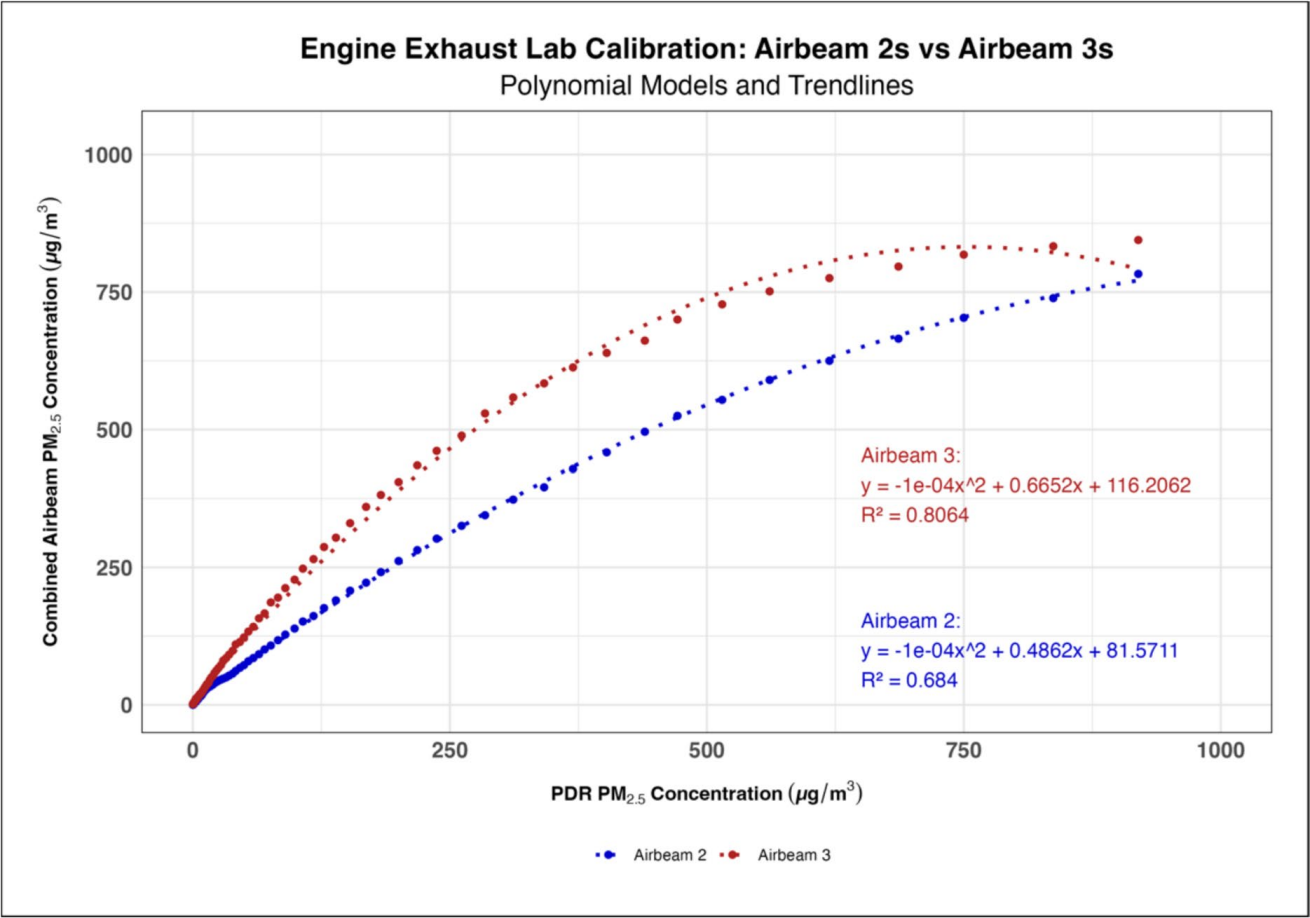
Polynomial regression model showing how the AirBeam 3s had a higher calibration coefficient on average compared to the AirBeam 2s when measuring engine exhaust

**Fig. 4:**
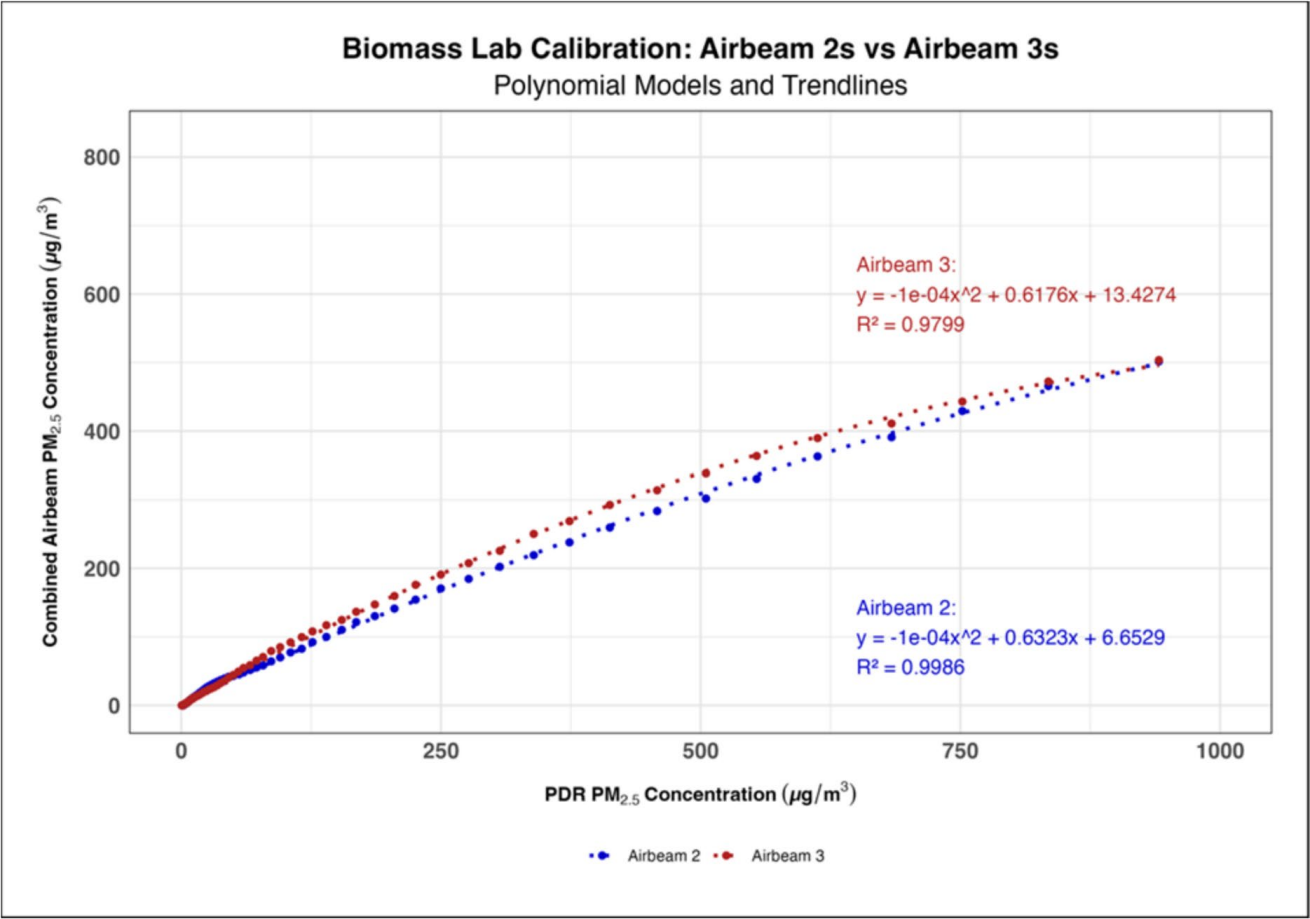
Polynomial regression model showing how the AirBeam 3s had slightly lower calibration coefficients and *R*^2^ values than the AirBeam 2s when measuring biomass smoke

Time series generated for each AirBeam 3 (Supplementary Figure S2) showed that the AirBeam 3s’ did not experience the measurement anomaly the AirBeam 2s demonstrated where they measured PM_1_ as being higher than PM_2.5_; thus, they did not need to be reprogrammed prior to use. When measuring engine exhaust, the AirBeam 3s had higher calibration coefficients on average compared to the AirBeam 2s with a mean linear coefficient of 0.192 and 0.102 for each generation of AirBeams, respectively (Fig. 1). However, when measuring biomass smoke the AirBeam 3s had slightly lower calibration coefficients compared to the AirBeam 2s with their respective mean linear coefficients being 0.274 and 0.323 (Fig. 2). The polynomial regression models consistently had higher *R*^2^ values than the linear regression models in both the engine exhaust and biomass smoke lab calibrations indicating that the polynomial models were a better fit (Figs. 3 and 4). When comparing the engine exhaust and biomass smoke lab calibrations, the *R*^2^ values were slightly higher in the biomass smoke calibrations suggesting that the AirBeam’s measurements of biomass smoke were more consistent. In the biomass lab calibrations, there was a noticeable drop in *R*^2^ values for the AirBeam 3s between the first and second biomass calibrations with the *R*^2^ value generally being higher in the first calibration than the second calibration (Supplementary Table S3).

Reverse calibration coefficients which can be applied to the AirBeam’s measurements to get a more accurate approximation of the true PM concentration when measuring at high occupational concentrations were also calculated for both generations of AirBeams when measuring both types of aerosols. When measuring engine exhaust, the reverse calibration coefficients from the linear and polynomial models were 2.97*x* and −0.0083*x*^2^ + 8.27*x*, respectively, for the AirBeam 2s, while they were 2.73*x* and 0.0055*x*^2^ + 1.98*x*, respectively, for the AirBeam 3s. When measuring biomass smoke, the reverse calibration coefficients from the linear and polynomial models were 2.51*x* and 0.004*x*^2^ + 0.02*x*, respectively, for the AirBeam 2s, while they were 2.56 and 0.0064*x*^2^ + −0.75, respectively, for the AirBeam 3s (Supplementary Figures S3-S6). 

**Fig. 5:**
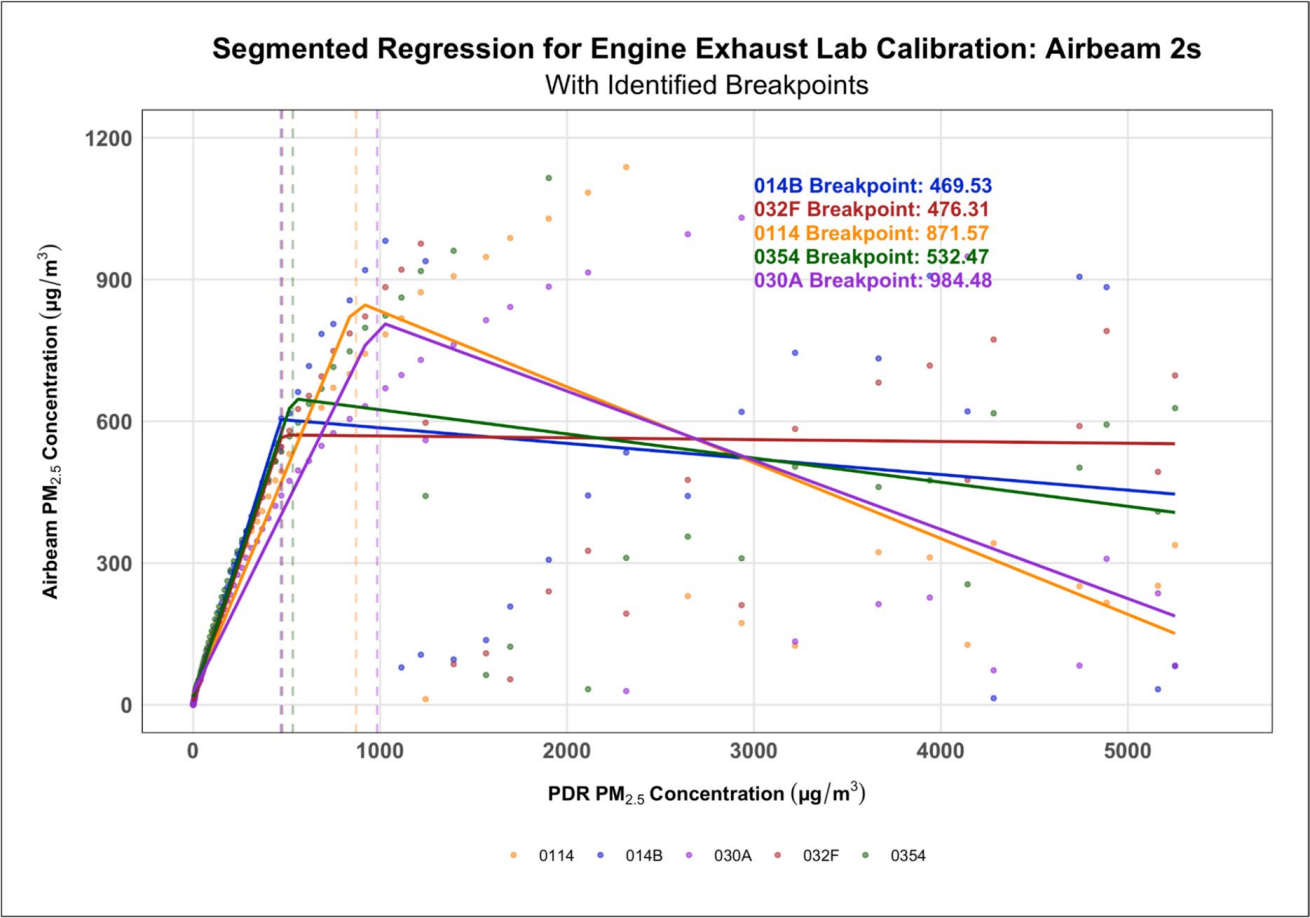
Segmented regression model showing how the AirBeam 2s generally had higher breakpoints than the AirBeam 3s during the engine exhaust calibration

**Fig. 6:**
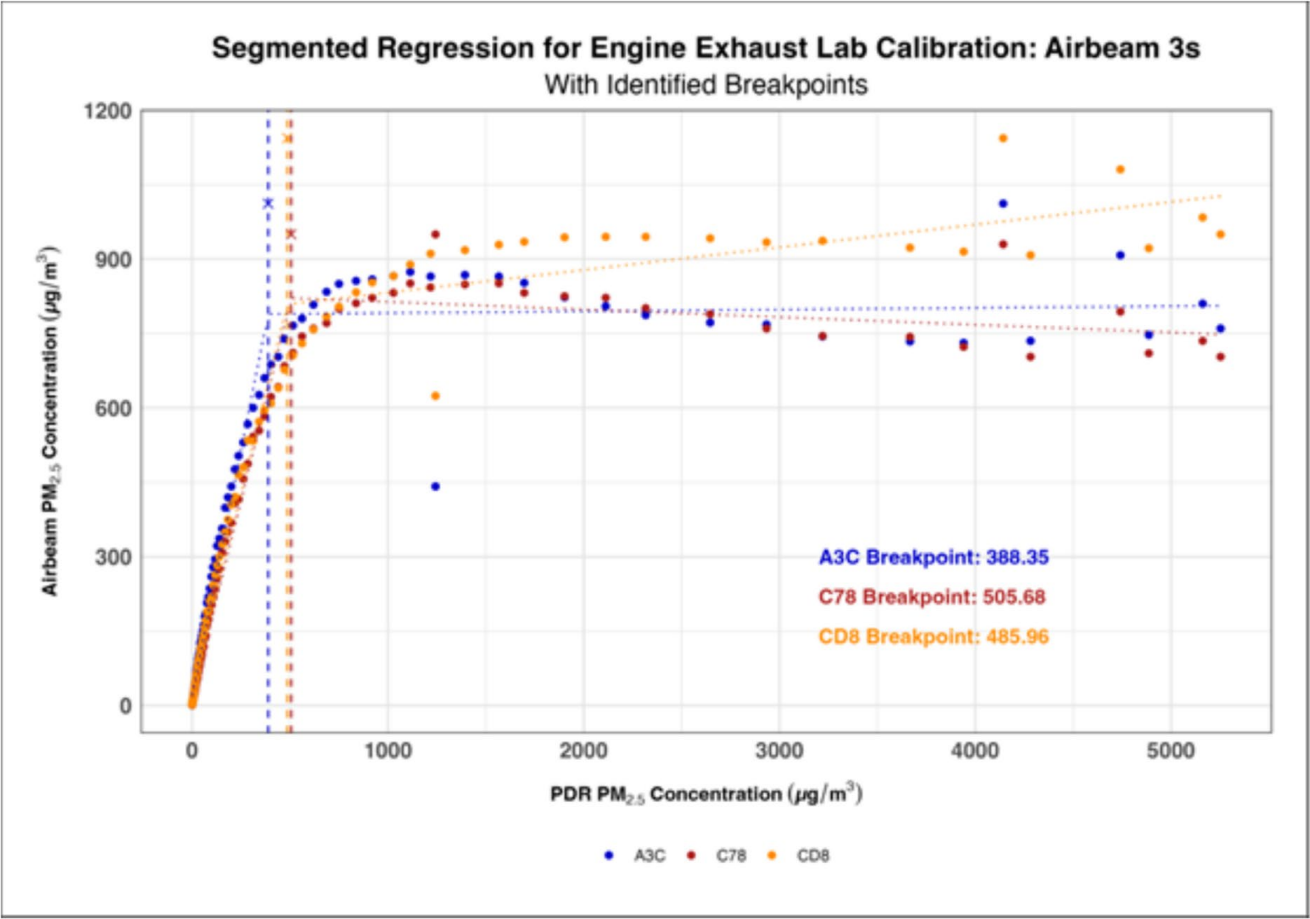
Segmented regression model showing how the AirBeam 3s generally had lower breakpoints than the AirBeam 2s during the engine exhaust calibration

Segmented regression plots generated for lab calibrations with each PM source (engine exhaust and biomass smoke) demonstrated that the reprogrammed AirBeam 2s tended to have higher breakpoints than the AirBeam 3s (Tables 2 and 3). However, the results also demonstrated that the break points varied significantly between PDR 1500s, between calibrations, and between PM sources (Figs. 5 and 6, Tables 2 and 3, and Supplementary Figures S7-S10). Despite this, there was a consistent range in which each generation of AirBeam’s breakpoints occurred suggesting that there was some uniformity in sensor behavior within each calibration and source of PM. At the same time, Figs. 5 and 6 highlight notable inter-instrument variability among individual AirBeam units, with breakpoints spanning from 469.53 to 984.48 µg/m^3^ in the AirBeam 2s and from 388.35 to 505.68 in the AirBeam 3s indicating meaningful performance differences even within the same sensor model.

**Table 2:** Comparison of breakpoints for AirBeam 2s and AirBeam 3s against PDR in the biomass smoke lab calibrations

**AirBeam #**	**Calibration 1 AirBeam breakpoint (PDR 1)**	**95% CI**	**Calibration 1 AirBeam breakpoint (PDR 2)**	**95% CI**	**Calibration 2 AirBeam breakpoint (PDR 1)**	**95% CI**	**Calibration 2 AirBeam breakpoint (PDR 2)**	**95% CI**
AirBeam 2s								
014D	1154.8 µg/m^3^	[1109.0, 1213.7] µg/m^3^	1100.9 µg/m^3^	[1046.6, 1156.9] µg/m^3^	2211.6 µg/m^3^	[1842.88, 2580.32] µg/m^3^	2354.7 µg/m^3^	[1878.59, 2830.81] µg/m^3^
030A	1124.7 µg/m^3^	[1073.7, 1175.6] µg/m^3^	893.6 µg/m^3^	[840.9, 946.2] µg/m^3^	2637.8 µg/m^3^	[2418.72, 2856.84] µg/m^3^	2544.4 µg/m^3^	[2264.84, 2823.96] µg/m^3^
0354	1114.9 µg/m^3^	[1063.7, 1166.1] µg/m^3^	1035.0 µg/m^3^	[978.7, 1091.4] µg/m^3^	2066.9 µg/m^3^	[1625.83, 2508.04] µg/m^3^	2272.7 µg/m^3^	[1775.23, 2770.17] µg/m^3^
AirBeam 3s								
A3C	783.5 µg/m^3^	[747.0, 820.0] µg/m^3^	626.8 µg/m^3^	[592.0, 661.5] µg/m^3^	1812.6 µg/m^3^	[1678.98, 1946.13] µg/m^3^	2286.1 µg/m^3^	[2078.26, 2494.0] µg/m^3^
C78	811.5 µg/m^3^	[776.0, 847.1] µg/m^3^	692.6 µg/m^3^	[657.4, 727.8] µg/m^3^	1977.4 µg/m^3^	[1872.82, 2081.91] µg/m^3^	2133.0 µg/m^3^	[1916.39, 2349.6] µg/m^3^
CD8	860.0 µg/m^3^	[822.0, 898.0] µg/m^3^	743.5 µg/m^3^	[706.7, 780.3] µg/m^3^	2033.3 µg/m^3^	[1869.38, 2197.13] µg/m^3^	2771.4 µg/m^3^	[2372.75, 3170.1] µg/m^3^

**Table 3:** Comparison of breakpoints for AirBeam 2s and AirBeam 3s against PDR in the engine exhaust lab calibration

AirBeam #	AirBeam breakpoint vs PDR in lab calibration	95% confidence interval
AirBeam 2s		
030A	984.5 µg/m^3^	[818.50, 1150.47] µg/m^3^
0354	532.5 µg/m^3^	[418.03, 646.92] µg/m^3^
014B	469.5 µg/m^3^	[329.63, 609.43] µg/m^3^
032F	476.3 µg/m^3^	[357.33, 595.28] µg/m^3^
0114	871.6 µg/m^3^	[731.22, 1011.91] µg/m^3^
AirBeam 3s		
A3C	388.4 µg/m^3^	[358.4, 418.3] µg/m^3^
C78	505.7 µg/m^3^	[481.2, 530.2] µg/m^3^
CD8	486.0 µg/m^3^	[452.9, 519.0] µg/m^3^

Finally, the tenfold cross-validation comparing the linear vs polynomial (quadratic) models for the AirBeam 2s and 3s showed that during the engine exhaust calibration for the AirBeam 3s, the polynomial model improved root mean squared error (RMSE) by ~ 8% and reduced bias, indicating a better fit, while for AirBeam 2s, RMSE improved by ~ 5%, but bias worsened slightly (Table 4). Thus, we recommend using linear models below the breakpoint, and polynomial fits above if RMSE improves by ≥ 5–8% without substantial bias increase when measuring engine exhaust. For the biomass calibration, the polynomial models slightly outperformed linear models for both the AirBeam 2s and 3s with RMSE improving by ~ 6–8%, with modest changes in bias. The AirBeam 2s showed lower mean absolute error (MAE) and minimal bias in the polynomial model, while the AirBeam 3s had high overall error in both models, but the polynomial model slightly reduced MAE (Table 4). Therefore, we recommend using polynomial models for biomass calibration when RMSE improves by ≥ 5% without a major increase in bias.

**Table 4:** Cross validation comparing the linear vs polynomial models for the AirBeam 2s and 3s in both the engine exhaust and biomass smoke lab calibrations

Metrics	Engine exhaust		Biomass	
	**Linear**	**Polynomial**	**Linear**	**Polynomial**
AirBeam 2s				
RMSE	1034.432 µg/m^3^	982.952 µg/m^3^	180.335 µg/m^3^	168.652 µg/m^3^
MAE	646.882 µg/m^3^	675.298 µg/m^3^	105.865 µg/m^3^	86.086 µg/m^3^
Bias	−0.540	−2.825	−0.672 µg/m^3^	−7.931 µg/m^3^
AirBeam 3s				
RMSE	876.570 µg/m^3^	809.196 µg/m^3^	3454.986 µg/m^3^	3443.398 µg/m^3^
MAE	602.382 µg/m^3^	474.598 µg/m^3^	1755.595 µg/m^3^	1440.884 µg/m^3^
Bias	−1.500	0.446	−78.941 µg/m^3^	−14.32 µg/m^3^

### AirBeam 2 field calibrations

**Fig. 7:**
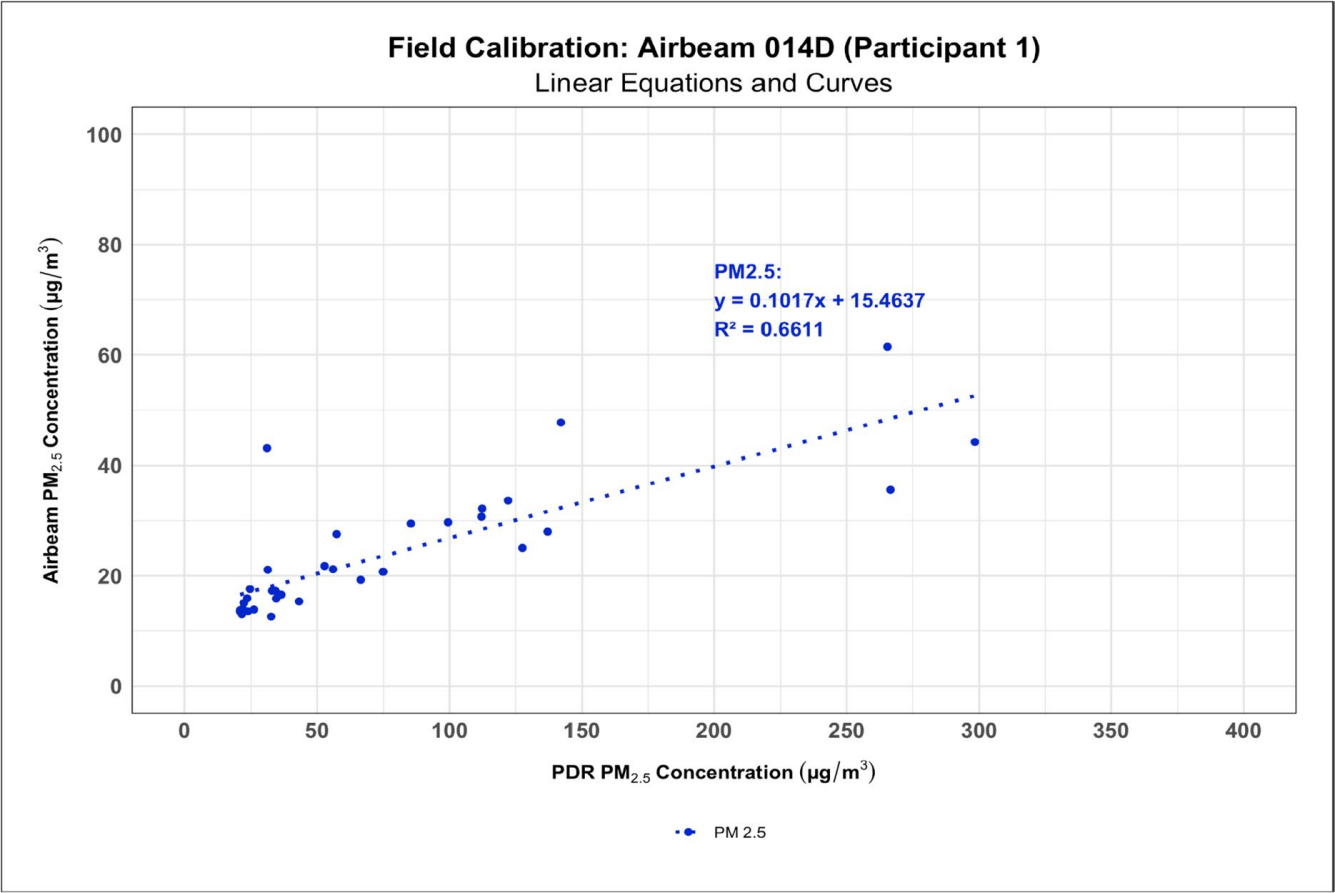
Linear regression model showing how the AirBeam 2 was not as sensitive to variations in PM concentration and generally recorded lower means than the PDR 1500 in the field

**Fig. 8:**
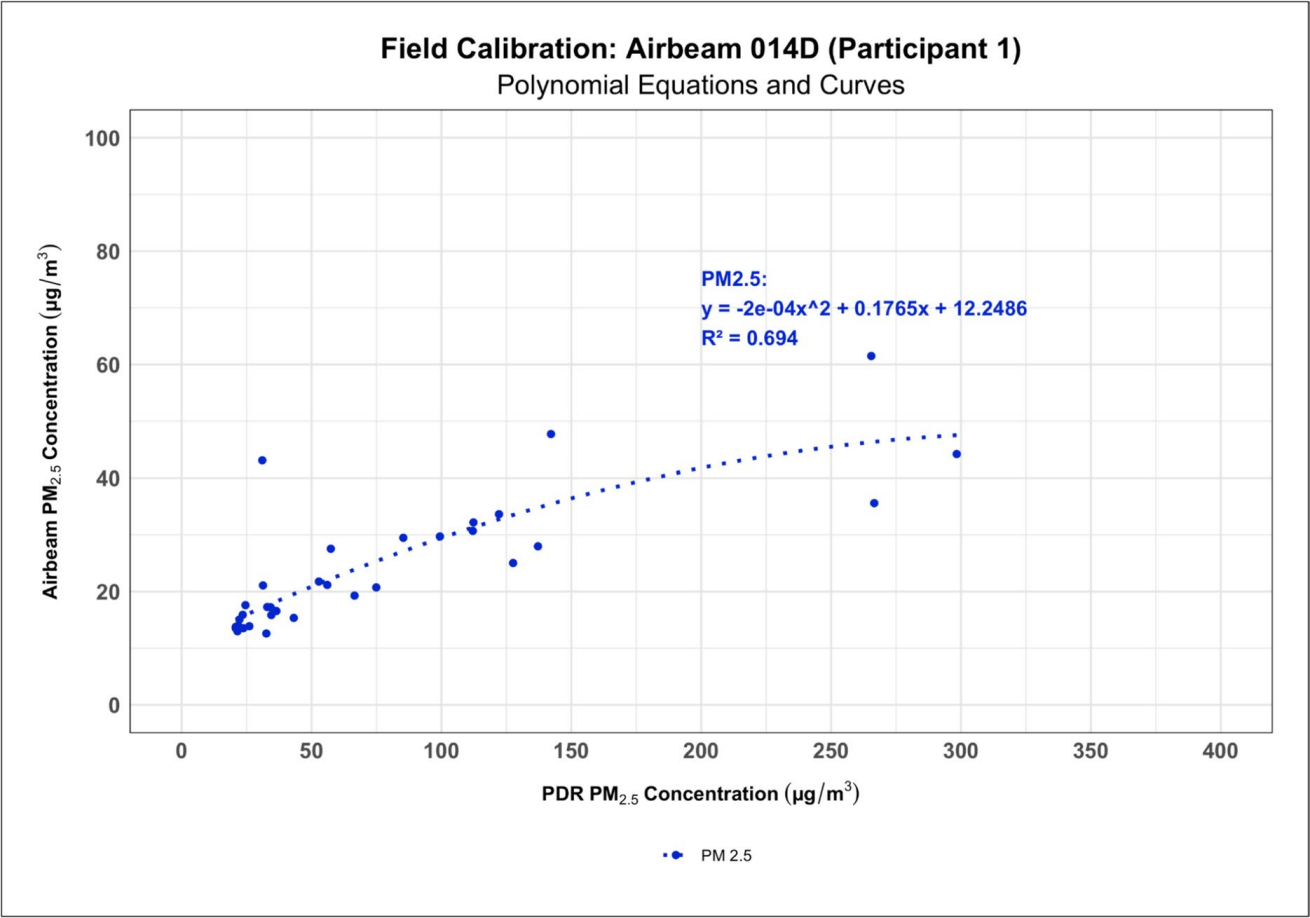
Polynomial regression model showing how the AirBeam 2 was not as sensitive to variations in PM concentration and generally recorded lower means than the PDR 1500 in the field

Linear and polynomial regression models generated from the field calibrations of the AirBeam 2s demonstrated that in occupational settings the AirBeam 2s were not as responsive to variations in PM concentrations as the PDR 1500. On average, the polynomial regression models had higher *R*^2^ values between the AirBeam 2’s and the PDR 1500’s PM_2.5_ measurements compared to the linear regression models indicating a better overall model fit. However, *R*^2^ values varied widely ranging from 0.0930 to 0.9218. In addition, these models also showed that the AirBeam 2s generally recorded lower mean PM concentrations than the PDR 1500 (Figs. 7 and 8, and Supplementary Figures S11and S12). The Supplementary Figures also show that the AirBeam 2s experienced reliability issues during the field calibrations where they would die or disconnect from the phones they were streaming their data to despite being nearby (participants held them in their pockets during the measurements) resulting in a loss of data. Supplemental Table S4 summarizes data completeness for each field calibration, including total measurement duration, valid minutes recorded, percent completeness, primary causes of data loss, and dropout based on falling below the 80% completeness threshold. For the sensitivity analysis, re-estimating the calibration metrics using only field calibrations sessions with ≥ 80% data completeness slightly increased RMSE (53.9 → 65.5 µg/m^3^, +21%) and MAE (26.0 → 35.7 µg/m^3^, +37%), while bias remained near zero and the slope decreased marginally (1.18 → 1.11, *Δ* = −0.07). These shifts are within expected variability for field co-locations and indicate that the calibration relationship is stable to moderate data loss. The results support that the model’s accuracy and sensitivity are not materially affected by session completeness above 80%, confirming robustness to typical LCS deployment gaps.

The results of the survey on participants’ device preferences showed that participants typically preferred to wear the AirBeam over the PDR 1500 due to its smaller size and lower impact on worker behavior. The survey also showed that prior to being given information about each of the devices, participants tended to trust the PDR 1500 more than the AirBeam due to its size and the perception that it housed more advanced equipment as a result. Similarly, after learning about each of the devices, the participants still tended to trust the accuracy of the PDR 1500’s measurements more due to it having advanced sensors and a physical cut point cyclone. However, participants tended to favor using the AirBeam because it was smaller and less laborious to carry, could measure multiple size fractions of PM, and could stream its data to wireless devices. Supplementary Table S5 shows participants’ response to the survey on device preference.

## Discussion

The results of this study underscore the importance and effectiveness of validating low-cost particulate matter (PM) sensors, such as the AirBeam, prior to their deployment for occupational PM measurements. During our initial laboratory calibration of the ten AirBeam 2 units, we observed an unexpected and previously undocumented measurement pattern where all ten AirBeam 2s consistently measured PM_1_ concentrations as being higher than PM_2.5_ concentrations above 50 µg/m^3^. To our knowledge, this is the first study to have experienced and identified this issue with the AirBeam 2. It remains unclear whether this issue is limited to a subset of devices within this generation of AirBeam 2 sensors or if it is a broader characteristic that has yet to be documented. However, it is highly possible that this was a symptom of measuring high PM concentrations outside of the manufacturer’s calibration range for this device, given it was designed to measure regular ambient PM concentrations. Fortunately, this issue was easily rectified by reverting to the original Plantower sensor’s default internal algorithms.

Despite some limitations, subsequent lab calibrations demonstrated strong sensor performance with high *R*^2^ values indicating a robust correlation between the reprogrammed AirBeam 2s’ measurements and the PDR 1500’s measurements below a device specific range. However, similarly to prior studies, we also observed inter-instrument variability between the AirBeam 2s and plateauing of the AirBeam 2’s measurements at higher concentrations (Feinberg et al., 2018; Kelly et al., 2017; Vercellino et al., 2018), further highlighting the importance of comprehensive validation of these low-cost sensors prior to their use in occupational settings. These findings are consistent with prior laboratory and ambient field studies that reported strong correlations with reference instruments under moderate concentrations but noted diminished accuracy or plateauing at higher concentrations (Karagulian et al., 2019; Kelly et al., 2017; Kim et al., 2019; Lim et al., 2019). Interestingly, we observed that the pre-reprogramming AirBeam 2s recorded fewer erroneous measurements at high PM concentrations than the reprogrammed AirBeams; however, after removing the erroneous measurements, the reprogrammed AirBeam 2s were more accurate and had a higher *R*^2^ value with the PDR 1500’s measurements than the pre-reprogramming AirBeam 2s, demonstrating that the optimal solution for addressing the AirBeam 2’s measurement issues is likely one that incorporates the strengths of both the manufacturer’s internal algorithms and the Plantower sensor’s default internal algorithms while addressing each of their weaknesses.

During the field calibrations of the AirBeam 2s, we only observed moderate correlations between the AirBeam 2s’ and the PDR 1500’s measurements, though these calibrations could have potentially been impacted by wind, outdoor humidity variations, and temperature. Unfortunately, given the highly variable nature of the weather in the Southeastern United States, we were unable to control for outdoor temperature and humidity variations in our study design. Thus, the AirBeam 2s likely have some limitations that may make them less effective at measuring PM concentrations in the field. Additionally, we encountered several reliability issues with the AirBeam 2s in the field, though our sensitivity analysis, where we re-analyzed the results of the field calibrations using only calibrations with 80% data completeness or more, showed that our findings were robust to missing data. These reliability issues included limited battery life with no way to monitor battery percentage and Bluetooth connectivity problems, both of which led to multiple field calibrations being cut short and contributed to data loss, supporting the conclusion by Sousan et al. (2021) that the AirBeam 2 still has limitations affecting its reliability (Sousan et al., 2021). However, many of these issues, like the Bluetooth connectivity issues and the lack of battery percentage, can be easily addressed with a software update from the AirBeam’s manufacturer to ensure smoother app and device operation. Furthermore, these issues can also be mitigated in several ways. Users can utilize the AirBeam’s cellular network capability with a SIM card to avoid Bluetooth connectivity issues. Regular re-calibration after each deployment and keeping devices connected to battery banks during field measurements can prevent power-related data loss. Compared to the results of the study by Kaur and Kelly (2023a, 2023b), where the Alphasense OPC-N3 and Plantower PMS5003 (a similar sensor to the one found in the AirBeam 2) successfully captured high-concentration dust events in the Salt Lake Valley and tracked temporal patterns relative to reference monitors, our field results showed weaker agreement (Kaur & Kelly, 2023), which could have been the result of greater variability in the humidity, temperature, and PM concentrations in the occupational environments where we performed our field calibrations, or that there is high inter-instrument variability between the Plantower sensors.

The results of the survey on participants’ perceptions and preferences for each device revealed a notable gap between the two device types that could influence their adoption in workplace monitoring programs. While participants generally trusted the accuracy of the PDR1500 more, they often preferred to use the AirBeam 2 because of its smaller size, lighter weight, lower cost, ability to measure different size fractions of PM simultaneously, and its ability to stream data wirelessly. This perception gap underscores the role of wearability, usability, and cost in shaping worker acceptance. Participants’ preference for the AirBeam 2 over the PDR 1500 highlights the growing demand for low-cost PM sensors which can be reliably used to measure PM in occupational settings and emphasizes the need to continue advancing sensor technology to deliver both high accuracy and practical usability in smaller, lighter, and more cost-efficient devices. Moreover, the substantially lower cost of low-cost devices allows for the purchase and deployment of many more units than a single industry-standard instrument, enabling greater spatial coverage of occupational environments. This expanded coverage can provide more granular exposure data, helping to identify high-risk areas, guide targeted interventions, and ultimately mitigate exposures and reduce the burden of occupational illness and injury.

Notably, the AirBeam 3s did not experience the same issue as the AirBeam 2s where they measured the PM_1_ concentration as being higher than the PM_2.5_ concentration and therefore did not need to be reprogrammed. Similarly to the AirBeam 2s, the polynomial regression models appeared to fit the AirBeam 3’s measurements better than the linear regression models. While linear models are the standard models used for calibration studies on low-cost PM sensors, we chose to include both linear and polynomial models because the polynomial models provided better model fit than the linear models and highlighted the plateauing relationship between the AirBeams’ and the PDR 1500s’ measurements at high PM concentrations. Both generations of AirBeams showed strong correlations and had higher *R*^2^ values with the PDR 1500’s measurements (in both the linear and polynomial regression models) when measuring biomass smoke compared to when measuring engine exhaust, suggesting that the AirBeams may be better equipped to measure biomass smoke than engine exhaust, or that the conditions in the chamber were more consistent during those calibrations. However, during the biomass lab calibrations, the AirBeam 3s showed a noticeable decline in *R*^2^ values from the first to the second calibration, with the *R*^2^ generally being higher in the first round. A similar but less pronounced trend was observed for the AirBeam 2s, where *R*^2^ values showed slight reductions, possibly indicating better sensor stability over time. This pattern of higher agreement between devices for some PM sources than others aligns with prior evidence that the chemical composition and particle size distribution of aerosols can influence low-cost sensor performance, reinforcing concerns raised in earlier studies regarding sensitivity to particle properties (Feinberg et al., 2018; Kelly et al., 2017).

It is worth noting that the particle size distribution and chemical composition of the particles can vary between biomass smoke and engine exhaust. Both biomass smoke and engine exhaust tend to produce particles with a geometric mean diameter (GMD) typically in the nanoparticle range (< 1 µm) with the typical GMD of biomass smoke ranging between 100 and 500 nm (nm), while engine exhaust tends to comprise particles with a GMD < 200 nm (Carrico et al., 2016; Hosseini et al., 2010; Karjalainen et al., 2014; Levin et al., 2010; Li et al., 2015; Luo et al., 2015). Furthermore, while both biomass smoke and engine exhaust largely comprise organic molecules like hydrocarbons and black carbon, they differ in that biomass smoke tends to have higher concentrations of inorganic ions and volatile organic compounds, while engine exhaust tends to have higher concentrations of trace metals like zinc, iron, and lead (Cappa et al., 2020; Chernyshev et al., 2018; Deka & Hoque, 2015; Hudson et al., 2004; Luo et al., 2015; Ponczek et al., 2022; Yang et al., 2019). Moreover, the chemical composition of the particles in each aerosol can affect the refractive index of the particles and how well light scatters off of them, with black carbon, a common component in both, having a relatively high refractive index (Axmann et al., 2015; Brown et al., 2021; Luo et al., 2022). Therefore, it is possible that the small particle size and/or chemical composition of the particles in each aerosol impacted the AirBeam’s ability to accurately measure the PM concentrations in each aerosol. Additionally, the AirBeam 3 was capable of measuring high PM concentrations without recording erroneous measurements like the AirBeam 2 did at high PM concentrations.

The breakpoint analyses of both the AirBeam 2s and AirBeam 3s demonstrated that, on average, the AirBeam 2s had higher breakpoints (the concentration at which a notable change in sensors’ behavior/performance occurs) than the AirBeam 3s indicating that the AirBeam 3s were either more sensitive to variations in PM concentrations or that the AirBeam 2s were capable of measuring higher PM concentrations before reaching their breakpoints. In addition, both generations of AirBeams tended to have higher breakpoints during the biomass smoke calibrations compared to the engine exhaust calibrations, indicating that they may be better suited for measuring biomass smoke at high concentrations. Greater variation in breakpoints between devices of the same generation was observed during the engine exhaust calibrations, suggesting that the sensors may be more sensitive to the specific physical and chemical characteristics of engine exhaust PM or that there are differences in sensitivity between individual units. Based on the results of our cross-validation analysis, we recommend using linear models below the breakpoints and polynomial fits above if RMSE improves by ≥ 5–8% without substantial bias increase when measuring engine exhaust, and we recommend using polynomial models for biomass calibration when RMSE improves by ≥ 5% without a major increase in bias. While variations in the breakpoints were present between generations, devices, and PM sources, there was a general consistency within each PM source, which signifies that the devices are likely reliable within their intended calibration environments. However, there was still notable inter-instrument variability within each generation, demonstrating that there are meaningful performance differences even between devices of the same model. This inter-unit variability is consistent with prior research showing that low-cost particulate matter sensors, including both Plantower- and Shinyei-based sensors, often differ in calibration performance due to manufacturing tolerances, sensor drift, and component aging (Anastasiou et al., 2022; Giordano et al., 2021; Sousan et al., 2016). For occupational applications, this variability has important implications as, without device-specific calibration, exposure estimates may be biased or inconsistent across workers, potentially leading to misclassification of exposures or inadequate intervention decisions. Therefore, to mitigate these risks, calibration against reference instruments prior to deployment and transparent reporting of measurement uncertainty should be considered essential when deploying low-cost sensors in occupational settings.

Regarding sensor deployment, these breakpoints can provide users with an idea of the upper limit of their sensor’s measurement capabilities or, in other words, the concentration above which the sensor’s measurement accuracy begins to diminish. Furthermore, these breakpoints can also help users evaluate the performance of their sensors in a variety of environments and with varying sources of PM. In this study, the variation in breakpoints between generations demonstrates that the AirBeam 2s may be better suited for taking measurements in environments where higher PM concentrations are expected, while the AirBeam 3s may be better suited for taking measurements in environments where lower PM concentrations are expected. However, it is worth noting that, despite minor limitations, both generations of AirBeams were able to measure high PM concentrations relatively well. These findings are consistent with recent occupational field calibration studies in mining environments, which showed that, with proper calibration, low-cost sensors can measure occupational aerosols like coal dust with relatively strong agreement to reference devices (Amoah et al., 2023; Penchala et al., 2025; Zaid et al., 2024). Our results extend this evidence by demonstrating that, despite certain limitations, the AirBeams are also capable of measuring high occupationally relevant PM concentrations, reinforcing their potential role in exposure assessment across diverse and demanding environments. Finally, as mentioned above, differences in the chemical compositions of the PM sampled could have affected the way that light scatters off the particles and potentially caused either the over or underestimation of PM concentrations in both the low-cost air quality sensors as well as the more advanced sensors, and therefore, further investigation is warranted.

While it is important to monitor workers’ exposures, it is best to limit exposures in the first place through effective preventive strategies. Based on prior research, potential recommendations could include implementing engineering controls such as improved ventilation systems or portable air purification units to reduce airborne PM concentrations in the workplace (Felgueiras et al., 2022; Ma et al., 2025). In addition, the use of appropriate personal protective equipment like masks and respirators can provide an added layer of protection when exposures cannot be fully eliminated (Expert Consensus Task et al., 2022; Yang et al., 2023). Finally, administrative measures such as adjusting work schedules or rotating job tasks may help minimize the duration and intensity of exposures for individual workers (Choi et al., 2023; Yang et al., 2023). Together, these approaches highlight the importance of combining exposure monitoring with proactive measures to protect workers’ health.

This study had several strengths including the large number of AirBeams we tested, the rigorous pre-deployment calibration procedures we employed which enhanced our confidence in the AirBeam’s reliability, the field calibration of the AirBeams 2s in occupational environments, the inclusion of a user survey to assess participant preferences, and the evaluation of the AirBeams’ performance when measuring PM from varying sources. Despite its many strengths, this study also had several limitations, including the inability to evaluate additional PM sources or their chemical composition, the small sample size and restriction to a single generation of AirBeams (AirBeam 2s) in the field calibrations, the lack of comparisons across different occupational environments, and the inter-instrument variability observed within the same generation, which reduced confidence in the accuracy of the AirBeam measurements.

Future studies could potentially strengthen these findings by improving the chamber used to calibrate the devices, to allow for the controlled introduction of diverse PM sources, which would enable further investigation into how PM of varying chemical compositions can affect the light scattering capabilities of both low-cost PM sensors and more advanced PM monitoring devices. In addition, they could also evaluate how the chemical composition of PM varies between sources using analytical chemistry techniques. The sample size of the field calibrations could be expanded to gather more data, which could be used to control for potential confounding variables that may not have been addressed during this study. Finally, while rigorous comparison between occupational environments was not feasible in this study due to there being a limited sample of measurements in each occupational environment and issues with missing data that left some occupational environments incomparable to others, future studies can improve on this in several ways. First, a larger and balanced sample size of field calibrations in various occupational environments should be considered when possible. Potential occupational environments that could be studied for comparison include construction sites, wildfires, home renovations, commercial kitchens, mechanic shops, commercial farms, factories, and coal-fired power plants and others as appropriate. Future studies would also benefit from performing multiple calibrations in each test environment to increase the comparability between environments and to reduce the impact of confounders. Implementing stratified co-location of the low-cost and more advanced PM sensors by environment and enforcing minimum completeness thresholds by ensuring a minimum of 80% data completeness for all calibrations considered in their analysis would greatly increase the robustness of the findings. Following these guidelines in future studies will be helpful for improving data quality, accuracy, and for preventing data loss when using these devices in occupational exposure assessments.

## Conclusion

In this study, we identified a previously undocumented measurement limitation in the AirBeam 2, underscoring the importance of the manufacturer’s internal algorithms in low-cost PM sensors. Following reprogramming with the Plantower sensor’s default algorithms, the AirBeam 2s showed significantly improved calibration coefficients (ranging from 0.9628 to 1.4494) and demonstrated a strong correlation with the PDR 1500 up to approximately 1000 µg/m^3^. The AirBeam 3s consistently performed well across aerosol types, maintained a strong relationship with the PDR 1500 without requiring reprogramming, and showed higher calibration coefficients for engine exhaust, whereas the AirBeam 2s were more responsive to biomass smoke. Breakpoint analysis suggested that AirBeam 2s may be better suited for high-concentration environments, while AirBeam 3s are more sensitive at lower concentrations. Although field deployments revealed some underestimation of PM_2.5_ by the AirBeam 2s, likely due to environmental factors, participants favored them for their portability, wireless capabilities, and ease of use. Taken together, our findings indicate that with careful device selection, prior validation, and regular calibration, AirBeam sensors can be reliably used to measure occupational PM concentrations in select environments, offering a cost-effective and accessible option for real-time exposure assessment in occupational settings.

## Supplementary Information

Below is the link to the electronic supplementary material.

ESM 1 (DOCX 1.83 MB)

## Data Availability

Should they be interested, readers may reach out to the corresponding author for the data used in the analysis of this study. Given these datasets contain data that has not yet been analyzed, the files associated with the data analysis will be available upon reasonable request.

## Declarations

### Software used to generate figures

All figures were generated using R v4.4.3.
