# Supplementary material for: Assessing the efficacy of a low-cost air pollution monitoring device for environmental and occupational exposure assessments
Source: Environ Monit Assess. 2025 Dec 11;198(1):40. doi: 10.1007/s10661-025-14870-1 (PMC12698801; doi:10.1007/s10661-025-14870-1)
Supplement: Supplementary file 1 — (DOCX 1.83 MB) [file 10661_2025_14870_MOESM1_ESM.docx]

Supplemental


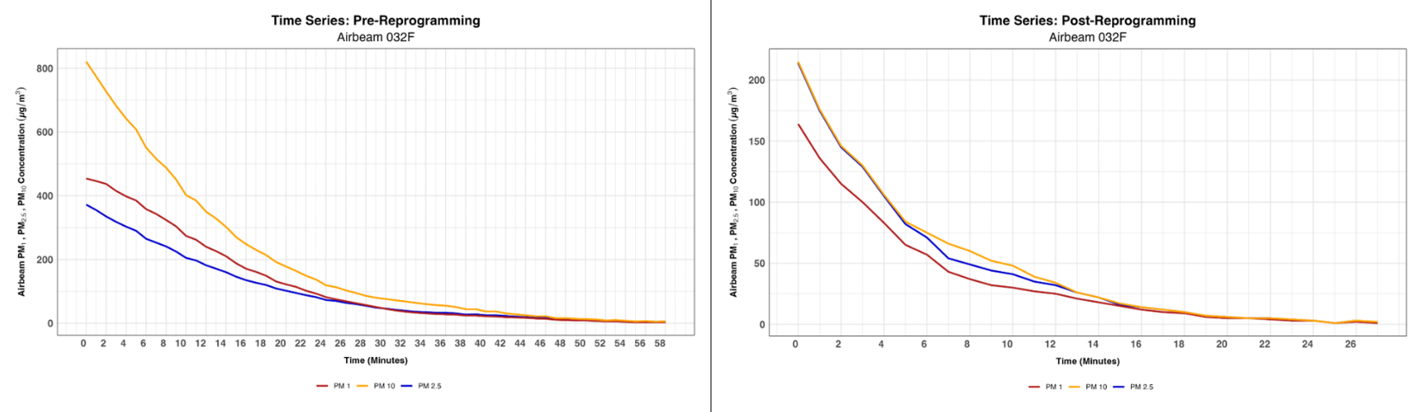

Supplementary Figure S1. Timeseries showing how the AirBeam 2 measured the PM_1_ concentration as higher than the PM_2.5_ concentration down to around 50 µg/m^3^ as well as how after reprograming the internal algorithms to the Plantower sensor’s default algorithms the AirBeam2 measured the PM_2.5_ concentration as being higher than the PM_1_ concentration but tracked very closely with the PM_10_ measurements.

**Detailed instructions on how to reprogram the AirBeam 2 to use the Plantower sensor’s default internal algorithm on PC:**

Windows PC instructions: 1) Download Arduino 1.0.6 via the Dropbox link: <https://www.dropbox.com/s/vlisuoun5x247a4/Arduino_AB2.zip?dl=0>. 2) Extract the files from the zip folder and install Arduino 1.0.6 on your computer. The following steps need to be repeated every time you reprogram the AirBeam 2. 3) Remove the AirBeam 2 screws and open the enclosure. 4) Launch Arduino IDE 1.0.6. 5) Via the Arduino IDE, navigate to Tools then Board and select [BootloaderCDC]AT90USB1286. 6) Note the COM ports listed in Arduino by navigating to Tools then Serial Port. 7) Turn on the AirBeam 2 and connect it to the PC via USB cable, then navigate to Tools then Serial Port and note the COM port associated with the AirBeam 2, this is the AirBeam 2 "run mode" COM port. 8) With the board oriented so the small metal box is facing you, press and hold down the left button, then press and hold down the right button. Hold both buttons for 5 seconds, then release the right button, then release the left button. Once you've done this there will be another COM port associated with the AirBeam 2, this is the AirBeam 2 "reprogramming mode" COM port. Select the "reprogramming mode" COM port in the Arduino IDE by navigating to Tools then Serial Port. 9) To upload the firmware, navigate to File then Upload or click the right facing arrow. 10) To revert to the default Plantower PMS7003 outputs, see "PMS_readings.cpp" lines 200-203 and update the equations based on lines 173-184.  To update PM_1_ delete "PM1_0 = 0.66776*pow(concPM1_0_amb, 1.1)" and replace it with "PM1_0 = concPM1_0_amb;", which is Plantower's default calibration equation for measuring PM1 in ambient air. 11) Once you have done this Power the AirBeam 2 off for 5 seconds then power it back on. Your unit is now reprogrammed with the updated firmware. You can now select the COM port associated with the AirBeam 2 "run mode" to see detailed information on the firmware you have uploaded. 12) Screw the enclosure back together.

**Detailed Quality Assurance/Quality Control checklist to mitigate the chance of data loss doing field measurements and calibrations:**To avoid loss of power during measurements, each AirBeam should be fully charged before use; for both the AirBeam 2 and 3, a full charge is indicated when the green charging light turns off while the device remains plugged in. Because charging time may vary between units, this approach provides the most reliable confirmation of a full charge. A portable charger may also be carried so that the AirBeam can be charged while measurements are being taken. To reduce the likelihood of data loss due to connection issues, use of a SIM card is recommended, as both AirBeam models allow you to insert a SIM card which enables them to log measurements through cellular networks. If Bluetooth must be used instead, the AirBeam should remain within a maximum of 5 feet (the closer the better) of the connected phone, and the connection should be checked at least every 15 minutes to ensure that data are still being streamed.

When performing calibrations, minimum co-location duration with a more advanced sensor may vary depending on the measurement environment and target concentrations. For field calibrations, a minimum co-location time of 30 minutes is recommended to ensure adequate comparison between the AirBeam and the reference instrument. For chamber calibrations, co-location should continue until the reference instrument reads at or near 0 µg/m³, noting that the required time will vary with the initial chamber concentration, as higher starting concentrations require longer clearing times.

To maximize measurement accuracy, AirBeams should ideally be recalibrated prior to each field deployment. If this is not possible, recalibration should occur at minimum once per week if the device is being used regularly. Finally, when analyzing data, only datasets with at least 80% completeness are recommended for use in drawing conclusions.

Supplementary Table S1. Calibration coefficients and R^2^ values from the reprogrammed AirBeams with the erroneous measurements, derived from linear and polynomial regression models.

|  | Linear Model | | Polynomial Model | | |
| --- | --- | --- | --- | --- | --- |
| AirBeam # | Post-reprogram coefficient with erroneous measurements | Post-reprogram R^2^ with erroneous measurements | Post-reprogram X^2^ coefficient with erroneous measurements | Post-reprogram X coefficient with erroneous measurements | Post-reprogram R^2^ with erroneous measurements |
| 014D | 0.91 (0.80 - 1.02) | 0.79 | -0.0011 (-0.0014 -0.0008) | 1.78 (1.51 - 2.05) | 0.871 |
| 0114 | 1.13 (1.05 - 1.21) | 0.93 | -0.0011 (-0.0012 -0.0010) | 2.00 (1.94 - 2.06) | 0.996 |
| 014B | 0.67 (0.48 - 0.85) | 0.41 | -0.0026 (-0.0030 -0.0022) | 2.72 (2.41 - 3.02) | 0.845 |
| 0138 | 0.64 (0.42 - 0.86) | 0.35 | -0.0027 (-0.0032 -0.0023) | 2.76 (2.39 - 3.13) | 0.803 |
| 030B | 0.74 (0.53 - 0.95) | 0.46 | -0.0027 (-0.0030 -0.0022) | 2.87 (2.64 - 3.10) | 0.932 |
| 0154 | 0.75 (0.56 - 0.94) | 0.51 | -0.0026 (-0.0030 -0.0022) | 2.62 (2.31 - 2.92) | 0.868 |
| 030A | 1.09 (0.96 - 1.21) | 0.89 | -0.0015 (-0.0017 -0.0013) | 2.16 (2.00 - 2.31) | 0.983 |
| 0161 | 1.17 (1.04 - 1.30) | 0.9 | -0.0015 (-0.0017 -0.0013) | 2.23 (2.07 - 2.39) | 0.984 |
| 032F | 0.65 (0.34 - 0.96) | 0.32 | -0.0036 (-0.0040 -0.0031) | 3.33 (2.88 - 3.60) | 0.905 |
| 0354 | 0.75 (0.58 - 0.93) | 0.52 | -0.0026 (-0.0030 -0.0022) | 2.01 (1.96 - 2.06) | 0.897 |

Supplementary Table S2. Shows the calibration coefficients from the polynomial regression model and the weights used to calculate the weighted average calibration coefficients for the reprogrammed AirBeam 2’s PM_1_ and PM_2.5_ measurements.

| **AirBeam** | **PM 1 Calibration Coefficient** | **PM 2.5 Calibration Coefficient** | **Number of Data Points** |
| --- | --- | --- | --- |
| 014D | 1.1214 | 1.7797 | 74 |
| 0114 | 1.4106 | 1.9969 | 63 |
| 014B | 1.2643 | 2.7172 | 74 |
| 0138 | 1.4644 | 2.7590 | 66 |
| 030B | 1.3832 | 2.8669 | 63 |
| 0154 | 1.1294 | 2.6166 | 63 |
| 030A | 1.6682 | 2.1552 | 41 |
| 0161 | 1.5431 | 2.2328 | 41 |
| 032F | 1.6655 | 3.2394 | 41 |
| 0354 | 1.3852 | 2.6699 | 69 |

**
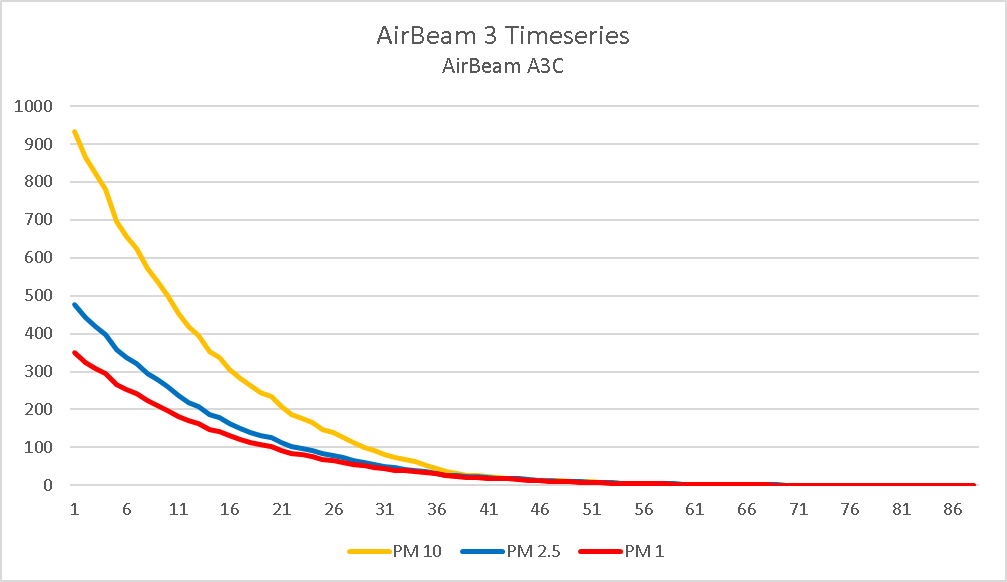
**Supplementary Figure S2. Timeseries of AirBeam 3 measurements with the manufacturer programmed internal algorithm showing how the AirBeam 3 did not experience the same measurement issue as the AirBeam 2 and therefore did not need to be reprogrammed prior to use.

Table S3: Polynomial R^2^ Comparison for Biomass Calibration 1 and 2 (AirBeam 3s Sensors)

| **AirBeam #** | **PDR** | **Polynomial Equation (Calibration 1)** | **R^2^ (Calibration 1)** | **Polynomial Equation (Calibration 2)** | **R^2^ (Calibration 2)** |
| --- | --- | --- | --- | --- | --- |
| **A3C** | **1** | y = -0.0001x^2^ + 0.635x + 15.625 | 0.975 | y = -1E-06x^2^ + 0.056x + 21.856 | 0.773 |
| **A3C** | **2** | y = -0.0001x^2^ + 0.669x + 17.578 | 0.968 | y = -6E-07x^2^ + 0.038x + 29.599 | 0.637 |
| **C78** | **1** | y = -0.0001x^2^ + 0.603x + 12.112 | 0.981 | y = -1E-06x^2^ + 0.049x + 16.831 | 0.831 |
| **C78** | **2** | y = -0.0001x^2^ + 0.635x + 13.925 | 0.975 | y = -6E-07x^2^ + 0.035x + 23.003 | 0.729 |
| **CD8** | **1** | y = -0.0001x^2^ + 0.615x + 12.545 | 0.983 | y = -1E-06x^2^ + 0.055x + 18.171 | 0.826 |
| **CD8** | **2** | y = -0.0001x^2^ + 0.647x + 14.406 | 0.978 | y = -6E-07x^2^ + 0.038x + 25.474 | 0.704 |


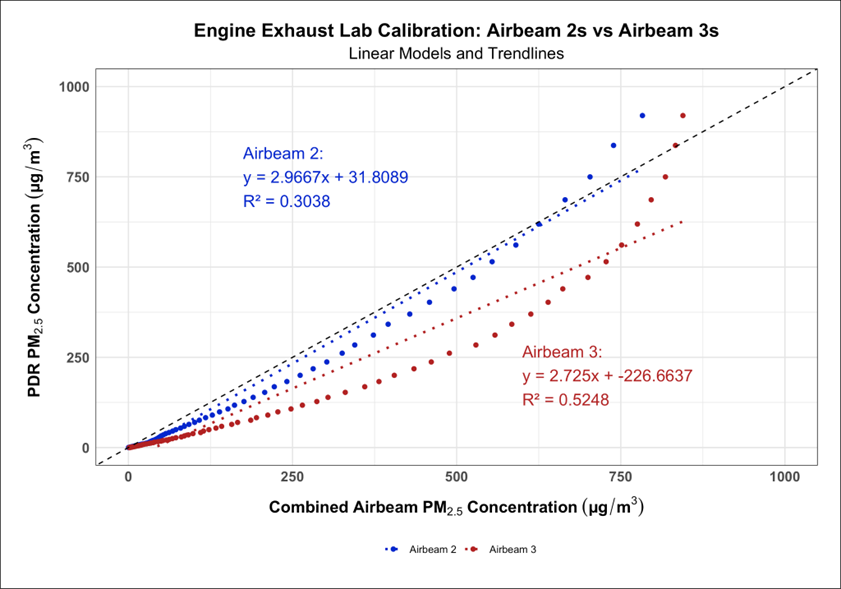

Supplementary Figure S3. Show the flipped linear regression model to calculate the linear correction factors for each generation of AirBeam when measuring engine exhaust.


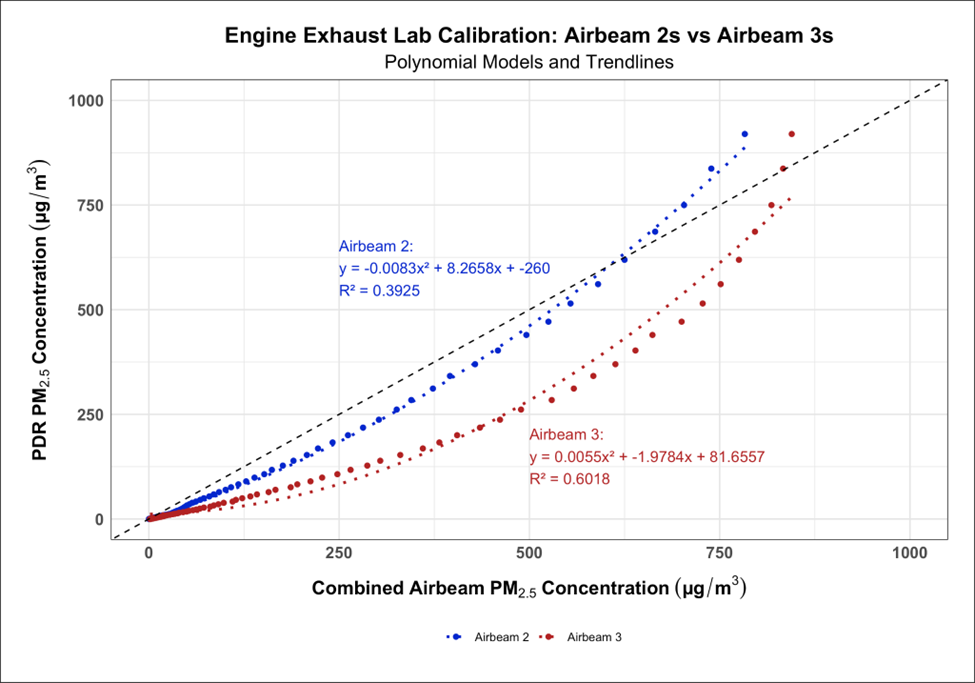

Supplementary Figure S4. Show the flipped polynomial regression model to calculate the polynomial correction factors for each generation of AirBeam when measuring engine exhaust.


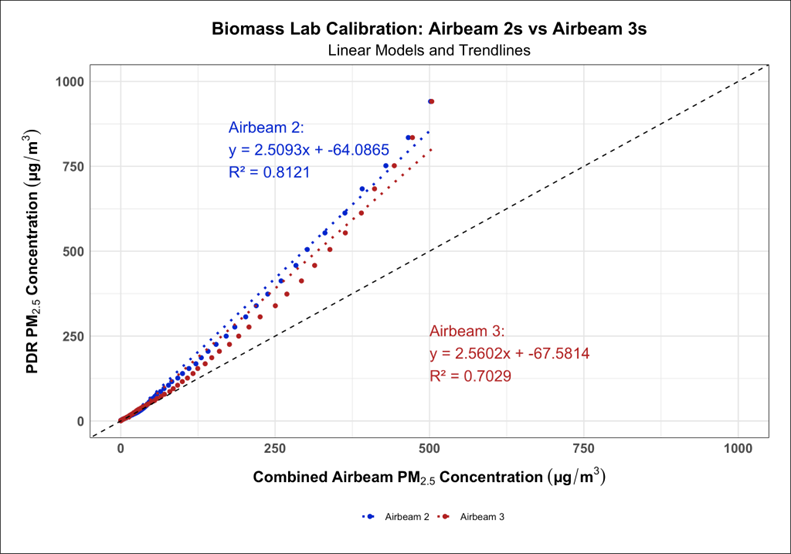

Supplementary Figure S5. Show the flipped linear regression model to calculate the linear correction factors for each generation of AirBeam when measuring biomass smoke.


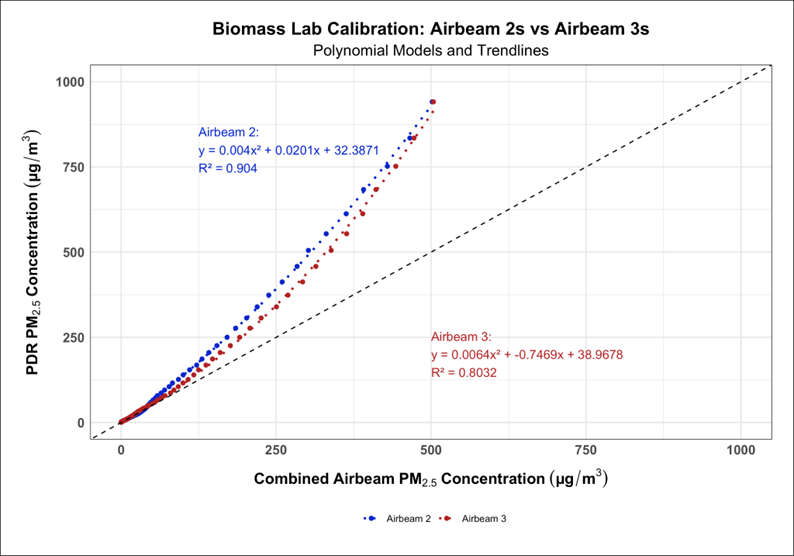

Supplementary Figure S6. Show the flipped polynomial regression model to calculate the polynomial correction factors for each generation of AirBeam when measuring biomass smoke.


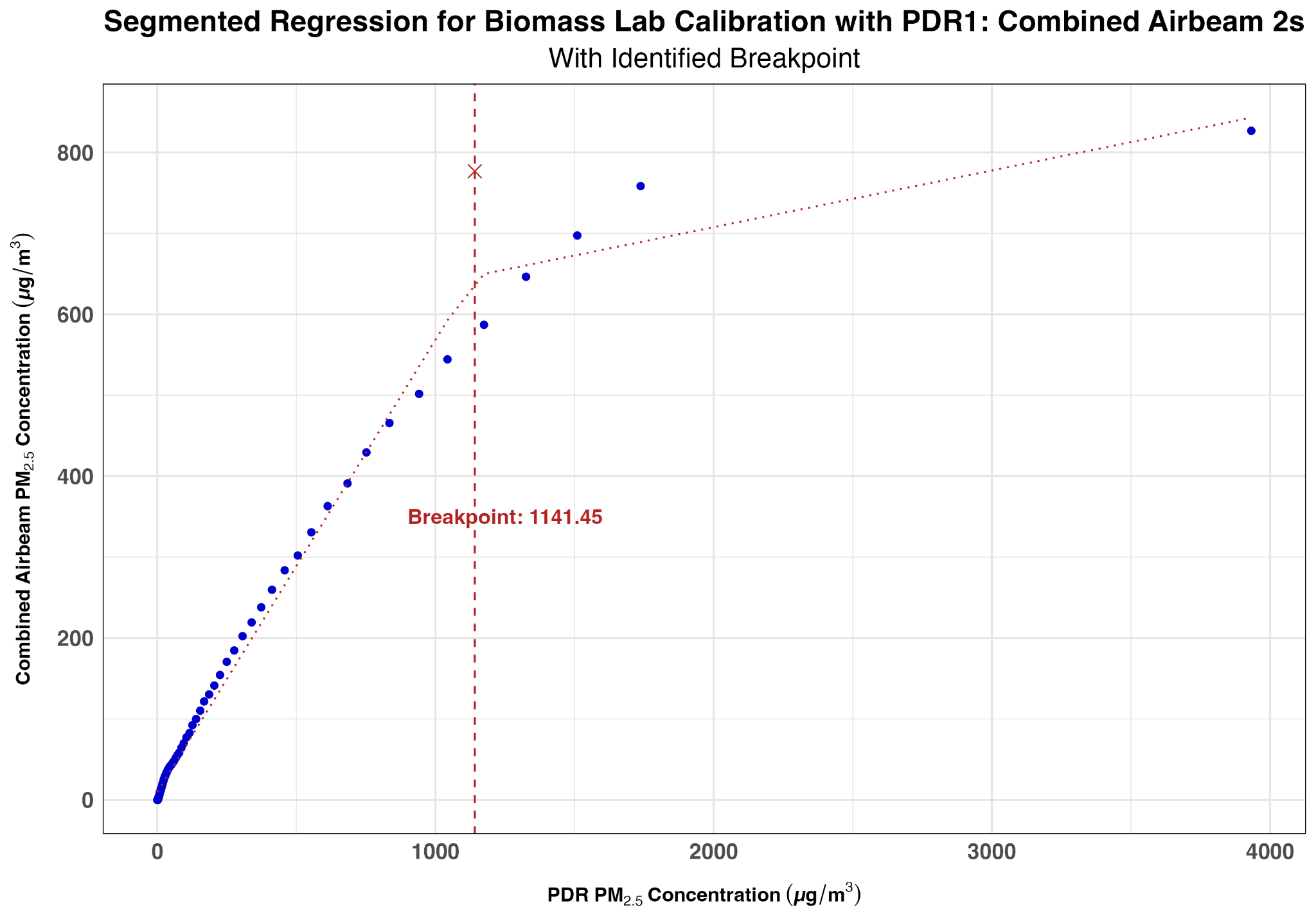

Supplementary Figure S7. Combined breakpoint for the AirBeam 2s with PDR 1 for the biomass lab calibration of the AirBeam 3s.


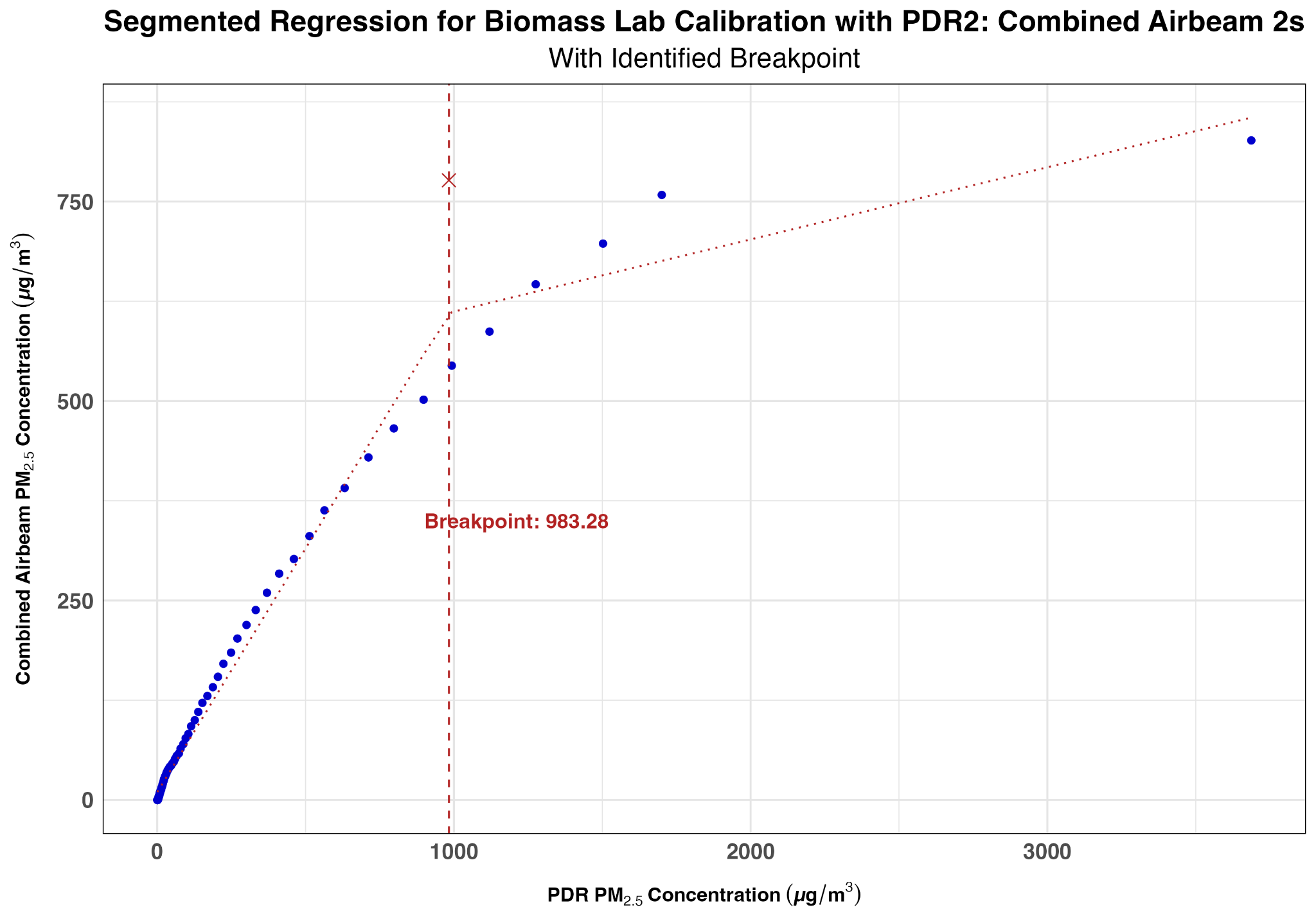

Supplementary Figure S8. Combined breakpoint for the AirBeam 2s with PDR 2 for the biomass lab calibration of the AirBeam 3s.


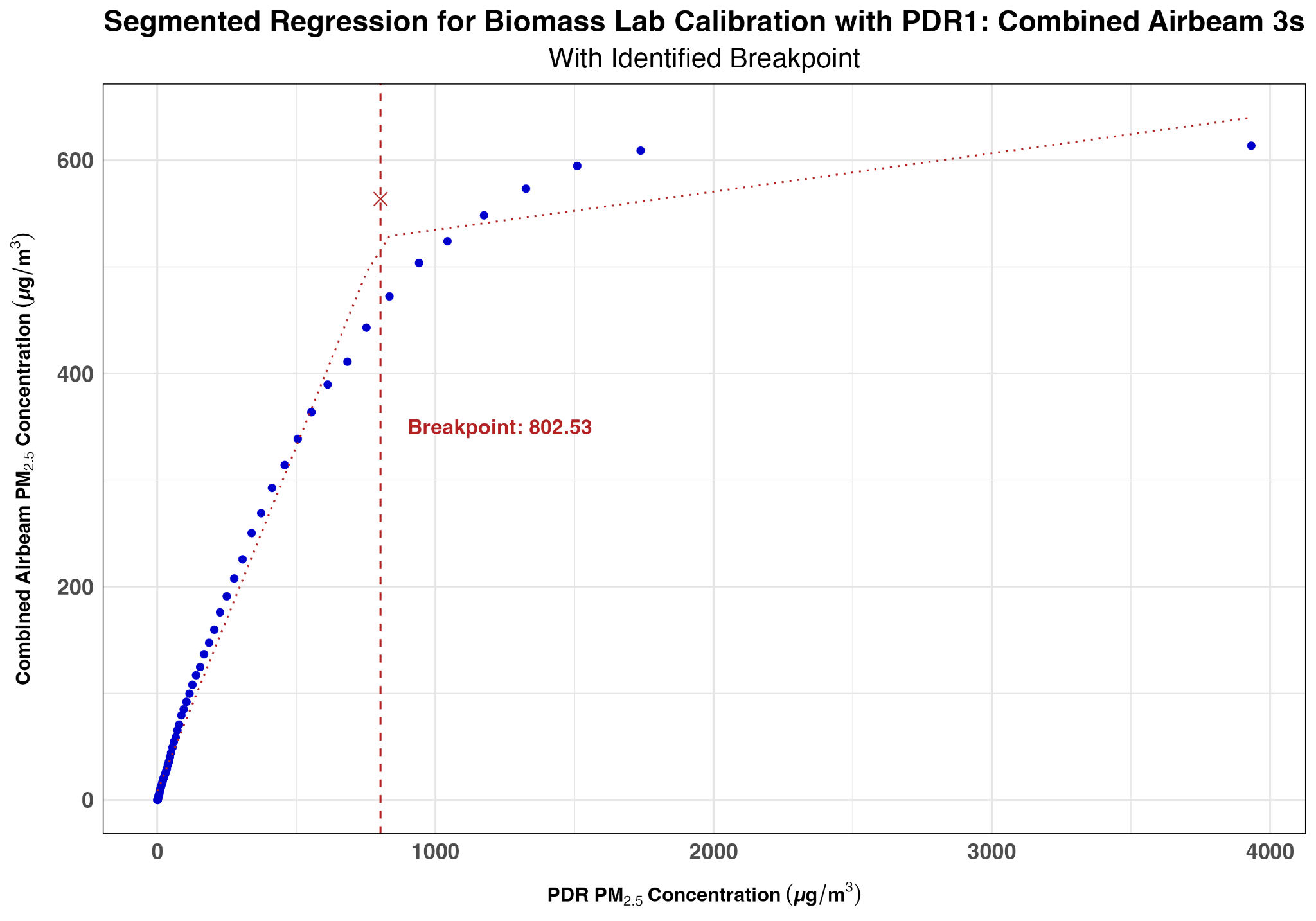

Supplementary Figure S9. Combined breakpoint for the AirBeam 3s with PDR 1 for the biomass lab calibration of the AirBeam 3s.


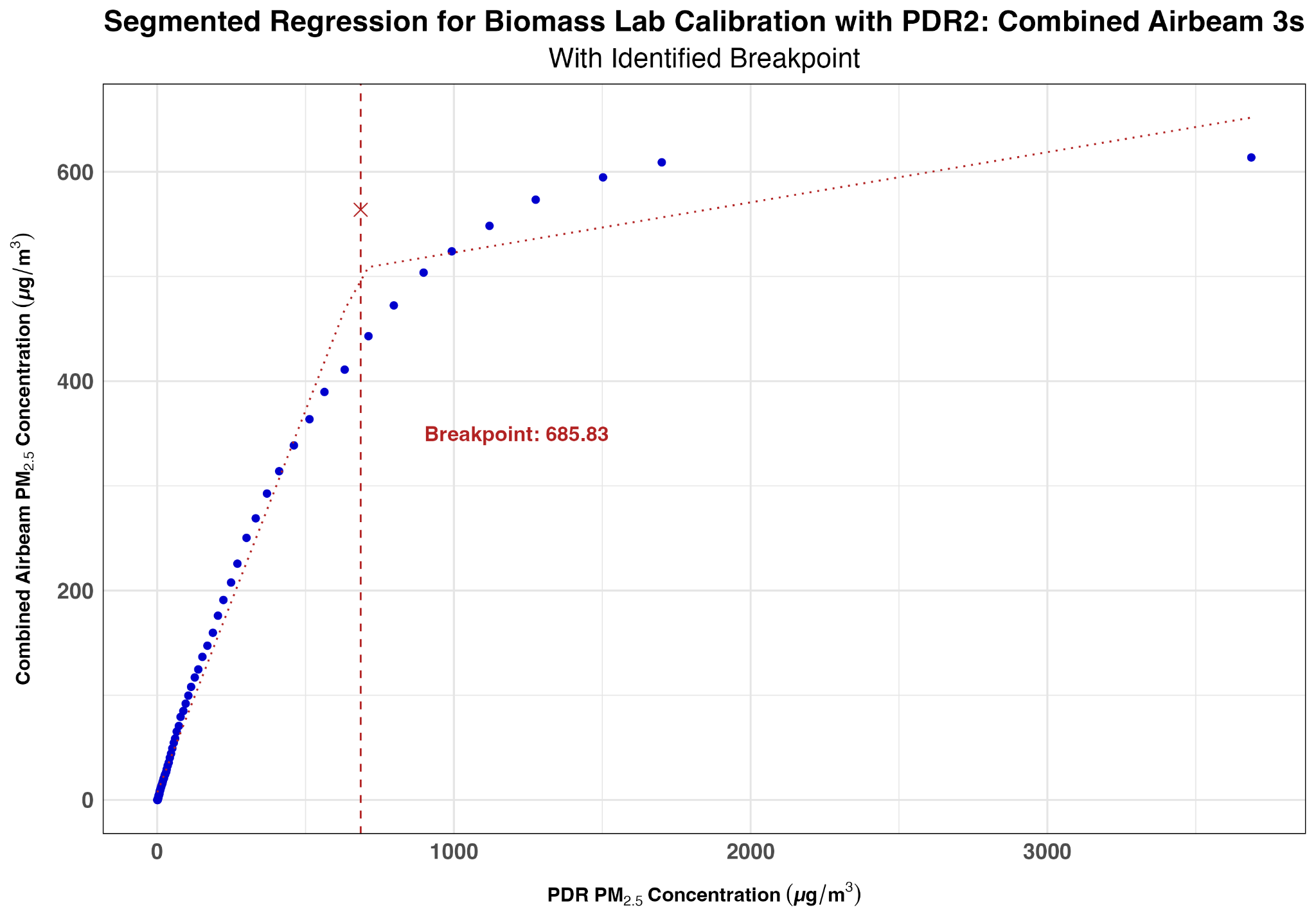

Supplementary Figure S10. Combined breakpoint for the AirBeam 3s with PDR 2 for the biomass lab calibration of the AirBeam 3s.


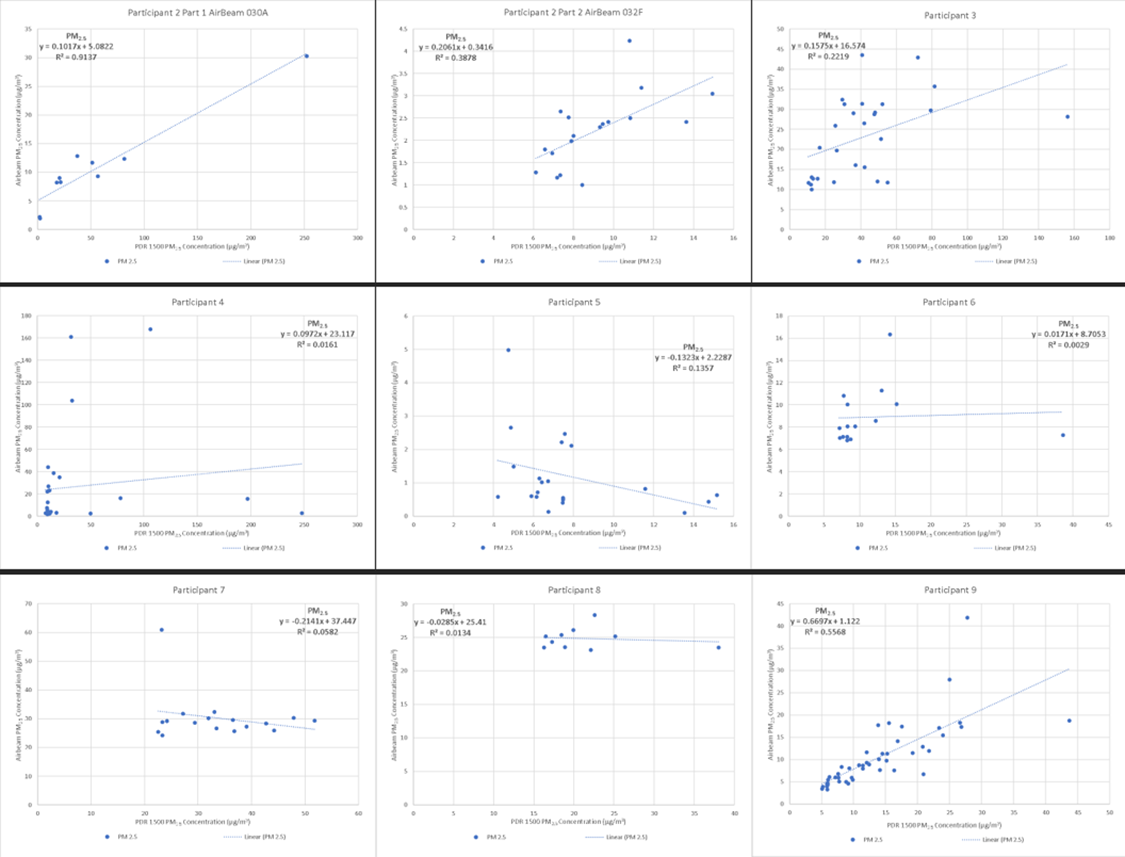
Supplementary Figure S11. Shows the linear regression equations and R^2^ values for the field calibrations of the AirBeam 2s against the PDR 1500 for participants 2-9.


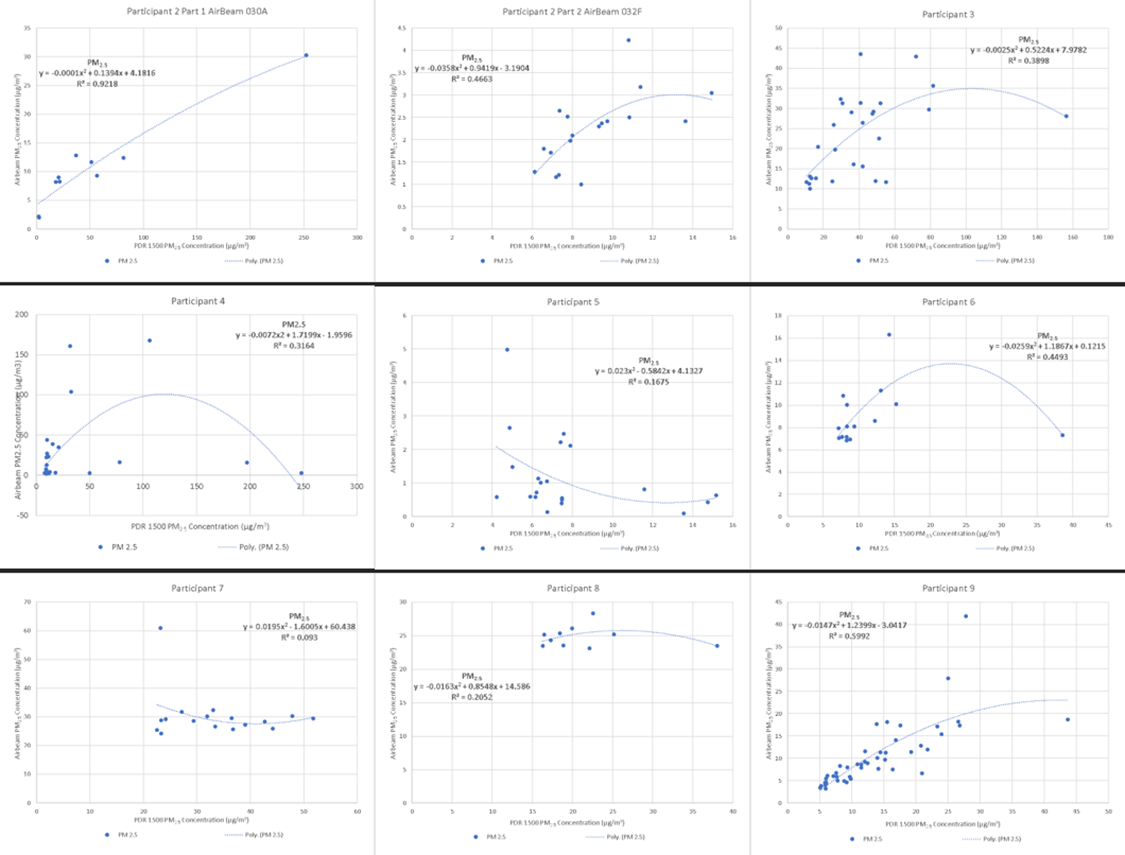

Supplementary Figure S12. Shows the polynomial regression equations and R^2^ values for the field calibrations of the AirBeam 2s against the PDR 1500 for participants 2-9.

Supplementary Table S4. Data completeness of field calibrations of the AirBeam 2s and primary cause of data loss

|  | **Total minutes** | **Valid minutes** | **Percent Completeness** | **Cause of lost data** | **Dropout** |
| --- | --- | --- | --- | --- | --- |
| **Participant 1**  **AirBeam 014D** | 38 | 38 | 100% | None | No |
| **Participant 2 AirBeam 030A** | 46 | 9 | 19.6% | Battery depletion | Yes |
| **Participant 2 AirBeam 032F** | 46 | 18 | 39.1% | None-Replaced AirBeam 030A after it died | Yes |
| **Participant 3 AirBeam 0138** | 30 | 28 | 93.3% | Bluetooth disconnected | No |
| **Participant 4 AirBeam 030A** | 30 | 27 | 90% | Bluetooth disconnected | No |
| **Participant 5 AirBeam 032F** | 30 | 21 | 70% | Battery depletion | Yes |
| **Participant 6 AirBeam 0138** | 30 | 15 | 50% | Bluetooth disconnected | Yes |
| **Participant 7 AirBeam 014B** | 30 | 17 | 56.7% | Bluetooth disconnected | Yes |
| **Participant 8 AirBeam 014D** | 31 | 10 | 32.3% | Battery depletion | Yes |
| **Participant 9 AirBeam 014B** | 46 | 46 | 100% | None | No |

Supplementary Table S5: Participants responses to survey on which device they preferred and trusted more:

| ID | 1 | 2 | 3 | 4 | 5 | 6 | 7 | 8 | 9 |
| --- | --- | --- | --- | --- | --- | --- | --- | --- | --- |
| Type of work | Driver and yard work | Yard work and environmental engineering | Maintenance tech, carpenter, painter and yard work | Yard work | Construction and remodeling | Freelance handyman | Land scaping and yard maintenance | Land scaping and yard maintenance | Lawn care, construction, EMT, ambulance driver |
| Which monitor did you prefer to wear? | AirBeam | AirBeam | AirBeam | AirBeam | AirBeam | AirBeam | AirBeam | AirBeam | AirBeam |
| Reason for preference | Smaller and unnoticeable when worn, PDR is not practical and heavy | Lighter and more compact | Lighter | Small and lighter, PDR is laborious | Lighter and unnoticeable when worn | Slimer and did not impede work | Smaller, lighter, and unnoticeable when worn | Lighter and less bulky | Lighter and less noticeable |
| Which monitor do you think is more accurate? | PDR 1500 | PDR 1500 | PDR 1500 | PDR 1500 | PDR 1500 | PDR 1500 | PDR 1500 | PDR 1500 | PDR 1500 |
| Why do you think it is more accurate? | PDR has better parts and more parts; size makes it appear more accurate | Bigger and seems to have more instruments for measuring | More expensive | Physical cut point for filtering by size rather than algorithm like AirBeam | Because it is bigger | More expensive and more advance instruments | Could hear it running, had a display, is bigger, and therefore should have more technology | Because it is bigger and therefore should be able to carry more sensor technology | More visible air intake |
| Which monitor was more comfortable to wear? | AirBeam | AirBeam | AirBeam | AirBeam | AirBeam | AirBeam | AirBeam | AirBeam | AirBeam |
| Have you ever worn a monitor before? | No | No | No | No | Yes | No | No | No | No |
| If yes, what type? | NA | NA | NA | NA | Don't know | NA | NA | NA | NA |
| Which monitor do you trust to provide better information on your exposure levels to air pollution? | PDR 1500 | PDR 1500 | PDR 1500 | PDR 1500 | PDR 1500 | PDR 1500 | PDR 1500 | PDR 1500 | AirBeam |
| Why do you trust it more than the other device? | Better sensors, cost more, measures bigger air | Industry standard device and has more research behind it. | Shows concentration during measurement | Price and physical cut point | It has a physical cut point and can adjust for environmental factors like humidity better | More complex devices with more advanced technology. | More expensive, bigger, and a physical cut point. | Because it has a physical cut point for particulate matter. | Easier to wear, less impact to work, gives estimate of all 3 sizes |
